# Electroactive Polymers for On‐Demand Drug Release

**DOI:** 10.1002/adhm.202301759

**Published:** 2023-11-12

**Authors:** Manal E. Alkahtani, Moe Elbadawi, Christopher A. R. Chapman, Rylie A. Green, Simon Gaisford, Mine Orlu, Abdul W. Basit

**Affiliations:** ^1^ UCL School of Pharmacy University College London 29–39 Brunswick Square London WC1N 1AX UK; ^2^ Department of Pharmaceutics College of Pharmacy Prince Sattam bin Abdulaziz University Alkharj 11942 Saudi Arabia; ^3^ School of Biological and Behavioural Sciences Queen Mary University of London London E1 4NS UK; ^4^ Department of Bioengineering Imperial College London London SW7 2AZ UK; ^5^ Centre for Bioengineering, School of Engineering and Materials Science Queen Mary University of London London E1 4NS UK

**Keywords:** additive manufacturing, controlled release, digital health, poly (3,4‐ethylenedioxythiophene), polyaniline, polypyrrole, voltage responsive

## Abstract

Conductive materials have played a significant role in advancing society into the digital era. Such materials are able to harness the power of electricity and are used to control many aspects of daily life. Conductive polymers (CPs) are an emerging group of polymers that possess metal‐like conductivity yet retain desirable polymeric features, such as processability, mechanical properties, and biodegradability. Upon receiving an electrical stimulus, CPs can be tailored to achieve a number of responses, such as harvesting energy and stimulating tissue growth. The recent FDA approval of a CP‐based material for a medical device has invigorated their research in healthcare. In drug delivery, CPs can act as electrical switches, drug release is achieved at a flick of a switch, thereby providing unprecedented control over drug release. In this review, recent developments in CP as electroactive polymers for voltage‐stimuli responsive drug delivery systems are evaluated. The review demonstrates the distinct drug release profiles achieved by electroactive formulations, and both the precision and ease of stimuli response. This level of dynamism promises to yield “smart medicines” and warrants further research. The review concludes by providing an outlook on electroactive formulations in drug delivery and highlighting their integral roles in healthcare IoT.

## Graphical abstract

Electroactive formulations promise to revolutionize medicines, offering unprecedented control over drug release. This review summarizes recent developments in the field, with a focus on conductive polymers (CPs). Furthermore, the potential of these digitalized formulations to be combined with other technologies, such as 3D printing, is also discussed. It is envisaged that CPs will transform personalized drug delivery.

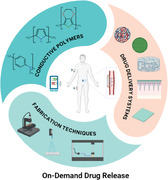

## 1 Introduction

The digitization of healthcare has created a paradigm shift in the way healthcare is delivered, offering new opportunities. While some sectors have adopted the change quite successfully, for example, in the development of remote monitoring technologies such as wearable diagnostic and sensory devices, other sectors need further consideration including drug development. Traditional drug delivery systems (DDS), such as those administered orally or in injectables, have significantly contributed to the treatment of diseases, having a significant societal benefit. To give a therapeutic effect, drug molecules need to be released from the DDS and become available for interaction with the body. The typical pattern of drug release involves diffusion, erosion, or swelling, which rely on the passive release of the drug, and hence are preprogrammed.^[^ ^1^ ^]^ In other words, once administered there is no further control over release characteristics.^[^ ^2^ ^]^ Most of the presently available DDS are not able to deliver the drug in the required dose, to the target site, and within a specific time. Some of the disadvantages of this approach include side effects and toxicities.^[^ ^3^ ^]^ Side effects and adverse drug effects are the most common concerns for medication safety. They mostly occur due to off‐target drug action or higher doses contributing to medication noncompliance and nonadherence, and such practice raises concerns.^[^ ^4^ ^]^ Thus, it is necessary to develop DDS with better safety and efficacy attributes. Furthermore, the discovery of new potent therapeutics such as biologics has increased the need for new, and sophisticated delivery systems. Understandably, a growing body of research has raised the importance of developing DDS with better control over drug release in which both the rate and the amount are precisely tailored to meet specific needs.

Recent advancements in material science have yielded state‐of‐the‐art materials with organized structures and high performance; one important example is stimuli‐responsive polymers. Such polymers have gained much interest from both academia and industry owing to their ability to mimic the behavior of living systems.^[^ ^5^ ^]^ Stimuli‐responsive polymers, also referred to as “smart” or “intelligent” polymers, exhibit chemical and/or physical alterations when subjected to internal or external stimuli.^[^ ^6^ ^]^ These systems could be sensitive to single, dual, or multiple stimuli.^[^ ^7^ ^]^ At the macromolecular level in the polymer chains, the changes may appear as bond cleavage, degradation, hydrophilic to hydrophobic equilibrium, and configuration.

**Figure 1:**
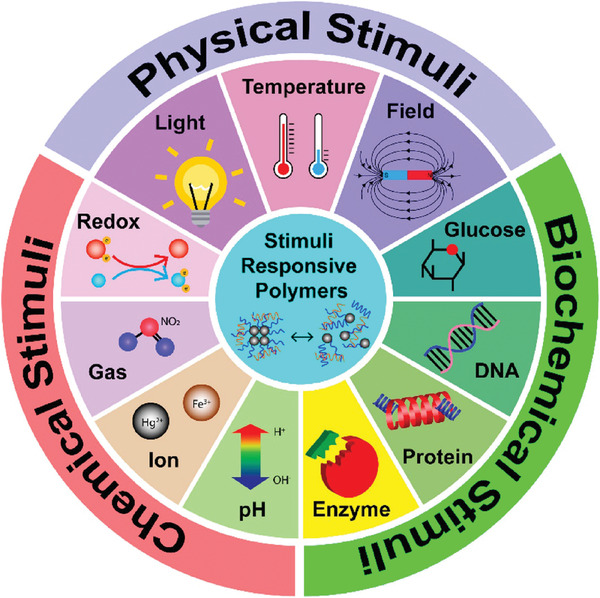
Different types of stimuli that trigger the release of drugs from stimuli responsive polymers. Reproduced with permission.^[^ ^13^ ^]^ Copyright 2021, American Chemical Society.

Based on the stimuli' nature, they can be classified into biological (e.g., enzymes and glucose), chemical (e.g., pH and redox), and physical (e.g., temperature, light, electrical field, and magnetic field),^[^ ^8^ ^]^ **Figure** **1** highlights more examples of stimuli types. Hydrogels, micelles, and polymer‐drug conjugates are classes of stimuli‐responsive systems that have been researched for drug, anticancer, oral protein, and gene delivery.^[^ ^9^ ^]^ Moreover, the release rate could be adjusted based on the changes in the delivery site microenvironment.^[^ ^10^ ^]^ These systems hold numerous advantages such as precise control of drug release rate and triggered tuneable and targeted delivery, which is particularly important for the delivery of chemotherapeutics, anti‐inflammatory drugs, psychotropic, and hormonal therapy.^[^ ^11^ ^]^ Accordingly, they reduce the risk of unwanted side effects associated with off‐target drug action and unnecessary high drug doses. Moreover, stimuli‐responsive DDS can diminish the harmful “dose‐dumping” effects of burst release.^[^ ^12^ ^]^

Electroactive materials are one class of stimuli‐responsive systems that are garnering attention, partly galvanized by the recent FDA approval of a conductive polymer (CP)‐based device.^[^ ^14^ ^]^ Electricity has indeed been harnessed by society since the 1800s, but it is worth mentioning that nature has exploited electricity for longer. One salient example is the use of “bioelectricity” for maintaining normal physiological functions, such as neuronal signaling and muscle contraction.^[^ ^15^ ^]^ In medicine, electric fields have been used in electroporation and iontophoresis to aid the drug molecules' transport through membranes. Electric fields have been used directly as well in the treatment of tumors.^[^ ^16^ ^]^ Thus, utilizing electric fields to trigger drug release from DDS is a tempting approach. For a DDS to be responsive to electrical stimulus, it must be conductive and allow electron transport.^[^ ^17^ ^]^ While blending polymers with conductive fillers, for example, silver nanowires (Ag‐NWs), carbon nanotubes (CNTs), and graphene can result in a conductive DDS,^[^ ^18^ ^]^ intrinsic CPs are a collection of polymers that are already inherently conductive.^[^ ^11^ ^]^ Conductive and electroactive are used interchangeably throughout this review to describe polymers with inherent conductive properties.

CPs have shown great potential to afford controlled and on‐demand drug release. Previous reviews have discussed the early developments of CPs and this review will provide recent developments in electroactive DDS.^[^ ^18a^ ^]^ The following section details the mechanism by which CPs are controlled, which applies to the different CPs detailed herein. Thereafter, recent progress is presented for three of the most studied CPs for drug delivery which are polyethylenedioxythiophene (PEDOT), polypyrrole (PPy), and polyaniline (PANi). Compared to other CPs, PEDOT, PPy, and PANi are the most commonly used in drug delivery owing to their unique electrical and physicochemical properties.^[^ ^19^ ^]^ They possess good biocompatibility, biodegradability, and low toxicity.^[^ ^20^ ^]^ In addition, they have high surface area, good charge storage capacity, and high conductivity which make them suitable for the controlled release of drugs.^[^ ^21^ ^]^ Moreover, they can be easily synthesized, and their properties can be tuned by changing the synthesis parameters. They are versatile and can be combined with other materials forming composites with tailored properties.^[^ ^22^ ^]^ This review will highlight approaches that have been explored to overcome previously reported issues with such systems, including reduced sensitivity to stimulus, low drug loading capacity, and poor mechanical properties.^[^ ^18a^ ^]^ Furthermore, the fabrication process of such systems is also discussed. The final section will discuss the future outcomes, and how electroactive DDS can be integrated with other emerging technologies to synergistically advance developments in the field.

## 2 Conductive Polymers (CPs)

The electrical stimulus is a type of physical stimulus that triggers the release of drugs from stimuli‐responsive systems. The material(s) comprising this system is electrically conductive to respond to an electrical stimulus, and accordingly release its load.^[^ ^23^ ^]^ CPs are inherently conductive; different examples of CPs and their advantages, limitations, and applications are highlighted in **Table** **1**. Nevertheless, not all of these were explored in the field of drug delivery for several reasons including complex synthesis, stability issues, and difficult processing. Yet, if they were investigated, they might open new opportunities for drug delivery.

**Table 1:** Common CPs and their properties, advantages, and limitations.

Conductive polymer (CP)	Acronym	Chemical structure^a)^	Conductivity [S cm^−1^]	Advantages	Limitations	Application	Reference
Poly (3,4‐ethylene dioxythiophene)	PEDOT	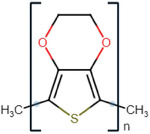	10^2^ – 10^3^	Stable High conductivity Biocompatible Water soluble	Low mechanical strength Complexity of synthesis	Drug delivery Neural prosthetics electrodes	[24]
Polypyrrole	PPy	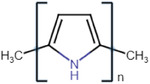	10^−3^ – 10^−1^	High conductivity Stable Biocompatible Mechanical strength	Water insoluble Brittle	Biosensors Drug delivery Tissue engineering	[25]
Polyaniline	PANi	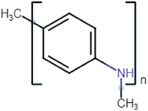	10^−4^ – 10^−2^	Stable Inexpensive High conductivity	Insoluble Low plasticity	Biosensors Drug delivery Tissue engineering	[26]
Poly (p‐phenylene vinylene)	PPV	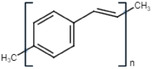	10^−4^– 10^−2^	Thermal stability High transparency	Water insoluble Lower conductivity (requires doping)	Biosensors Photovoltaic devices	[27]
Polythiophene	PTh	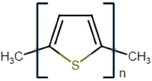	10–10^3^	High conductivity Biocompatible	Difficult to process. Water insoluble Instability	Biosensors Tissue engineering	[28]
Polyacetylene	PA	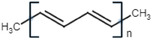	10^4^	High conductivity Chemical stability	Low stability Difficult to process	Chemical sensors Solar cells	[29]
Polycarbazole	PCz	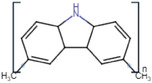	10–10^3^	Chemical stability Electron‐donating ability	Poor mechanical properties Complex synthesis Nonbiodegradable	Electronic devices Optoelectronic devices	[30]
Polypyridine	PyPy	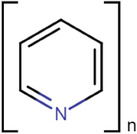	10–10^3^	Biocompatible High thermal stability	Poor mechanical properties Sensitive to humidity Nonbiodegradable	Electrochemical sensors Tissue engineering	[31]

^a)^ Chemical structures were drawn using chem‐space.

Among these, the most common inherently CPs are PEDOT, PPy, and PANi. CPs are also gaining interest in other fields, where they have been used to develop electrodes, biosensors, wearable electronics, sensors, monitoring devices, and robotics.^[^ ^19^, ^32^ ^]^ Thus, CPs have the potential to serve multiple roles in DDS. Some applications of CPs in the medical field include controlled drug release, nerve regeneration, and tissue engineering.^[^ ^33^ ^]^

As DDS, CPs provide programmable controlled release of drugs when subjected to an electrical stimulus, with the higher the applied voltage, the higher the drug release and vice versa.^[^ ^34^ ^]^ CPs are also known to be biocompatible, water‐soluble, highly conductive, mechanically sound, stable, and inexpensive, which are ideal properties for a DDS.^[^ ^35^ ^]^

### 2.1 Poly(3,4‐ethylenedioxythiophene)

Poly (3,4‐ethylenedioxythiophene) (PEDOT) is the most widely used CP due to its advantageous characteristics, such as excellent stability at ambient conditions, water solubility that allows its use for biological and medical applications, film‐forming properties, easy to process, low‐temperature processing and tuneable electrical conductivity that could be enhanced by the addition of either liquid or solid conductivity enhancers.^[^ ^36^ ^]^ PEDOT is typically doped with poly (styrenesulfonate) (PEDOT:PSS) to allow the CP to be dispersed in solvents.^[^ ^37^ ^]^ PEDOT:PSS‐based films final properties depend on the materials used within the films such as the solvent, the production method, and postproduction processing.^[^ ^14^, ^38^ ^]^

### 2.2 Polypyrrole

PPy has been extensively studied compared to other CPs due to its good intrinsic properties including high electrical conductivity and biocompatibility, low density, and ease of preparation.^[^ ^11^, ^39^ ^]^ This has led to the polymer gaining research attention in a wide variety of applications, such as neuronal grafts, artificial muscles, sensors, and microwave shielding.^[^ ^40^ ^]^ PPy can be produced by several fabrication routes, resulting in products such as nanoparticles, hydrogels, and films, with the latter achieving high conductivity of >380 S cm^−1^;^[^ ^41^ ^]^ whereas, nanoparticles and hydrogels achieve values in the order of 101 S cm^−1^.^[^ ^39^, ^42^ ^]^ Like PEDOT, PPy can be doped with PSS and other dopants to drastically improve its conductivity, thereby, expanding its performance.^[^ ^43^ ^]^

### 2.3 Polyaniline

PANi is another CP possessing great electrochemical conductivity, biocompatibility mechanical properties, and facile synthesis routes, first discovered 150 years ago.^[^ ^44^ ^]^ Interestingly, a degradable conductive scaffold can be achieved, further widening the application of PANi.^[^ ^45^ ^]^ It can be synthesized by a chemical oxidation process, and many different nanostructures of intricate designs can be produced. For example, altering the pH during oxidation can produce either nanofibrils or nanotubes.^[^ ^44c^ ^]^ The combination of PANi with other polymers as a blend, copolymer, or hydrogel has been exploited in sensors, and electronic devices, achieving conductivities of 10^−7^ to 10^−3^ S cm^−1^ in biomedical applications.^[^ ^44^, ^46^ ^]^

## 3 Drug Loading

**Figure 2:**
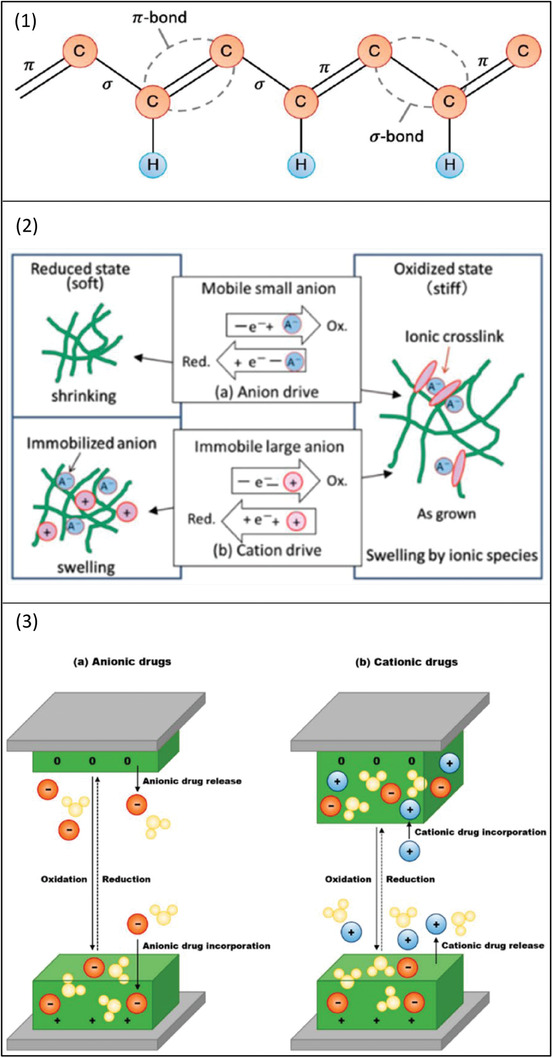
Schematic illustration showing 1) conductive polymers (CPs) conjugated chain 2) shrinking and swelling processes during redox reaction 3) mechanism of drug release for a) anionic drugs, b) cationic drugs. Reproduced with permission.^[^ ^15^, ^47^ ^]^ Copyright 2019, MDPI.

The inherent conductivity of CPs is the result of the polymer molecule comprising a conjugated chain of carbon–carbon single bonds (σ) and carbon–carbon double bonds (π) (**Figure** **2**(1)). When the p‐orbitals overlap in the π bonds, the electron movement between atoms becomes better and accordingly the electrons move within the polymer chain. When drugs are incorporated, they add or remove electrons to or from the polymer chain allowing their movement in and out based on the applied potential.^[^ ^15^, ^47^ ^]^

Drug incorporation can take place during the CPs polymerization, where anionic drug incorporation occurs by balancing the positive charges created during the CPs oxidation. Cationic drugs, on the other hand, are incorporated during the reduction process (Figure 2(2,3)). Despite being commonly used, the incorporation of drug molecules during polymerization could be reduced due to the interaction between drug molecules and polymers; in addition, drug molecules reduce the attachment of CPs into the electrode and consequently the system response to the electrical stimulus. Therefore, it is recommended that drug incorporation takes place after CPs synthesis.^[^ ^47^ ^]^ A key requirement of the drug is to be electrically charged, which includes anionic and cationic drugs. Being explored more frequently, anionic drugs are included within the system as a dopant during the monomer oxidation process.^[^ ^48^ ^]^ Nevertheless, this mechanism is associated with lowering the drug loading capacity, decreasing the conductivity, and both physical and mechanical properties. A dopant is a substance that is added in small concentrations to CPs to enhance their electrical conductivity. The method by which a dopant is introduced into a polymer is called doping. Doping could be chemical or electrochemical; in the former, the dopant molecules will be added to the polymer during the synthesis process, whereas, in the latter, a voltage will be applied to the polymer in the presence of a dopant solution. Based on the type and concentration of the dopant, and the doping method, the electrical conductivity of the polymer will be altered.^[^ ^49^ ^]^ An alternative incorporation method is to synthesize the CP with an anionic dopant, induce a reduction reaction to eliminate the dopant, and finally add the drug as a secondary dopant. Nevertheless, there is a risk of the drug undergoing a reduction reaction. Cationic drugs such as dopamine, chlorpromazine, and *N*‐methylphenothiazine have been entrapped in CPs, although less common than anionic ones. The two‐step method starts with the use of an anionic dopant and is followed by the reduction of the polymer and including electrons in its backbone.^[^ ^50^ ^]^

## 4 Mechanism of Drug Release from CPs

The mechanism of drug release from CPs depends on the alterations of the redox state (Figure 2 (2)). During the redox reaction, the polymer charge, volume, and conductivity undergo several changes.^[^ ^16^, ^51^ ^]^ In the reduction reaction, the CP volume is decreased leading to anion‐driven release of anionic drugs (Figure 2(3a)); whereas the opposite occurs during the oxidation reaction, the CP volume expands leading to the release of cationic drugs in a process called “cation‐driven actuation” (Figure 2(3b)). CPs volumetric expansion and reduction are controlled by altering the applied voltage.^[^ ^47^, ^49^ ^]^ Having a controllable and reversible redox reaction make CPs ideal for drug delivery.^[^ ^52^ ^]^

Drug release from CP‐based systems could be influenced by several parameters including the polymer thickness and density, the release media, and the form of electrical stimulation. Presuming that the thicker the polymer the higher the amount of drug incorporated, the amount of drug release does not seem to be linear with this. In fact, thicker films have lower electroactivity compared to thin ones, which negatively impacts the amount of drug release. Drug release media properties such as pH, ionic strength, polarity, and hydrophobicity have an impact on the CPs.^[^ ^53^ ^]^ Ion transport is affected by the media pH, anionic movements dominate in media with low pH values, and both cationic and anionic movements occur at neutral pH.^[^ ^54^ ^]^ The selection of the release media is mainly dependent on the physiological relevancy, for example, if the system is intended to deliver drugs to extracellular fluid, then a buffer solution with a pH value of 7.4 is applicable. As described earlier, the nature of electrical stimulation will impact the drug release as the redox state controls drug movement.^[^ ^48^ ^]^

The release process is a combination of electro‐chemo‐mechanical action in CPs that cause expansion and contraction movements leading to drug release. To initiate the drug release, different methods have been explored including the use of cyclic voltammetry (CV), chronoamperometry (CA), and chronopotentiometry. CV is an electrochemical technique for potentiodynamic measurement of redox reaction in materials but can be repurposed to actuate drug release. The test starts with a predetermined potential value and scan speed and is set to reach a certain potential value, then moves in the opposite direction until the initial potential is reached again. In CA, the potential of a working electrode is kept constant over time. Chronopotentiometry is a galvanostatic method in which a fixed current of the working electrode is set over a given time. In brief, if a higher voltage or longer activation time is employed, the drug release is expected to be greater.^[^ ^55^ ^]^ When a CV test is used, the electro‐chemo‐mechanical response of CPs films may cause mechanical inconsistency, which could be detected by scanning electron microscopy (SEM), which contributes to shortening the system life. These techniques could be coupled with some analytical techniques such as UV, and HPLC to quantify the release of drugs.^[^ ^18a^ ^]^

Neutral drugs, on the other hand, are more difficult to actuate. Different approaches have been explored, including hydrophobic–hydrophilic interaction between the drug and anionic dopant, generation of hydrogen bonds between the drug and oxidized polymer, and formation of covalent bonds between the drug and CP.^[^ ^19^, ^56^ ^]^ However, the drug release is primarily depending on intermolecular interactions and bond hydrolysis instead of responsiveness to external stimuli.^[^ ^55^ ^]^

Several studies have investigated different strategies to incorporate various drug types and explore diverse release mechanisms using different CPs; the following sections will discuss recent progress in specific CPs highlighting both drug incorporation and release mechanisms.

## 5 CPs in Drug Delivery

The distinctive attributes of CPs render them exceptionally appealing for a wide range of drug delivery applications. The subsequent sections will delve into the versatile landscape of CPs in drug delivery, examining their incorporation into diverse matrices, including hydrogels, polymeric films, nanoparticles, and more. Each section will elucidate the specific studies and applications where these CPs have been harnessed, highlighting the exciting potential they hold in modern pharmaceutical research and healthcare solutions.

### 5.1 Hydrogels

Hydrogels have emerged as versatile and promising drug delivery systems, offering a dynamic platform for precise and controlled release of therapeutic agents.^[^ ^57^ ^]^ These three‐dimensional networks of hydrophilic polymers possess the remarkable ability to absorb and retain large amounts of water or biological fluids while maintaining their structural integrity.^[^ ^58^ ^]^ This unique characteristic enables hydrogels to act as reservoirs, gradually releasing encapsulated drugs in response to various environmental cues.^[^ ^59^ ^]^ By tailoring their composition and properties, hydrogels can be engineered to respond to specific triggers.^[^ ^49^ ^]^ By incorporating CPs into hydrogel matrices, these “conductive hydrogels” have the ability to respond to electrical signals, enabling on‐demand drug release through electrochemical modulation.^[^ ^60^ ^]^

PEDOT:PSS can be used as a standalone electroactive DDS; however, its properties can be enhanced when blended with other polymers. For example, a DDS containing PEDOT as the CP and poly (dimethylacrylamide‐co‐4‐methacryloyloxy benzophenone (5%)‐co‐4‐styrenesulfonate (2.5%)) (PDMAAp) as a hydrogel precursor was developed by Kleber and team.^[^ ^61^ ^]^ The DDS demonstrated the successful active release of fluorescein, which was sixfold greater compared to a PEDOT:PSS system. This could be explained by the effect of the hydrogel the active drug release from the hydrogel system is faster than the standard system. The hydrogel system possessed the ability to reload and release the drug multiple times and the study demonstrated that fluorescein was reloaded using photolithographic techniques. Active fluorescein release from PDMAAp/PEDOT system was prompted using a potential of −0.5 V for 60 s which was followed by the reloading of fluorescein multiple times. The data showed that the amount of fluorescein release was maintained for three loading and stimulation cycles. The released amount of fluorescein upon the second cycle was 13.2 times higher than the control samples indicating the system's ability to reload and release the drug. There is a relationship between the applied voltage and the amount of fluorescein release; a current value of −0.6 V yielded 8.9 times more fluorescein release in comparison to −0.1 V. Different stimulation types result in different release profiles, for example, constant potentials provide strong initial burst release; single short pulses, on the other hand, will give a release profile a step‐like single profile. Lastly, CV sweeps will give a staircase‐like release.

**Figure 3:**
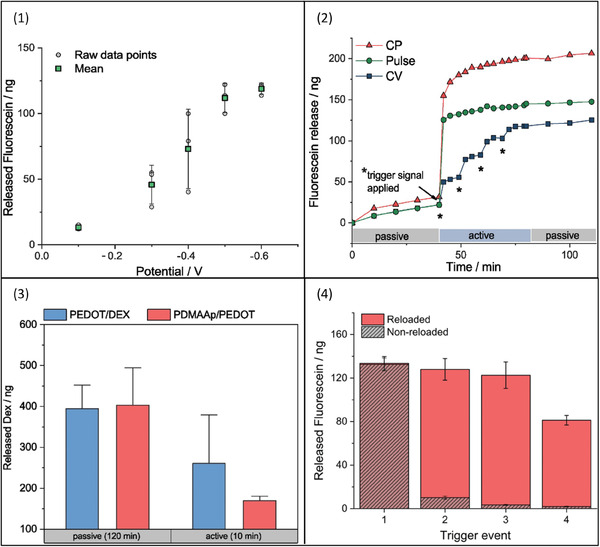
Release profiles under different conditions 1) flu release using different applied voltages. 2) passive and active release of flu using different stimulation types of CPs, pulse, and cyclic voltammetry (CV). 3) Dex release in both passive and active from PEDOT and PDMAAp/PEDOT formulations. 4) flu release from reloaded and nonreloaded formulations. Reproduced with permission.^[^ ^61^ ^]^ Copyright 2019, John Wiley and Sons.

The same study also demonstrated the active release of dexamethasone for local delivery to the neural interface. Dexamethasone has a similar charge and size as fluorescein, and it was used for its anti‐inflammatory activity, especially in the central nervous system. Both active and passive dexamethasone release studies were conducted on both PDMAAp/PEDOT and PEDOT/PSS systems. Passive release data showed that upon immersion of the systems for 120 min, 403.1 ± 61.0 ng, and 394.6 ± 38.5 ng dexamethasone released from PDMAAp/PEDOT and PEDOT/PSS, respectively. On the other hand, the active release results were 169.6 ± 7.2 and 260.7 ± 78.9 ng of dexamethasone were released from the PDMAAp/PEDOT and PEDOT/PSS, respectively, in 10 min (**Figure** **3**).

Hydrogels containing proteins were also successfully fabricated in an electroactive system. Gelatin methacryloyl (GelMA) were blended with PEDOT/*para*‐toluene sulfonate (*p*TS), where bovine serum albumin (BSA) was used as a model protein. The study demonstrated a significant increase in BSA release with a voltage of −0.6 V over 200 min. Furthermore, a 21‐day release study found that cycling the voltage between ±0.6 V at a frequency of 0.1 Hz for 1 h a day significantly altered the release rate compared to passive release. Thus, the application of an electrical stimulus was found to modulate the release rate and opens the opportunity to explore the potential modulating therapeutic protein delivery release over a prolonged period. It is worth noting that the addition of the conductive material enhanced the hydrogel stiffness when immersed in phosphate buffer saline solution (PBS) over a 21 day period and cell viability analysis revealed no adverse cytocompatibility effect with the addition of PEDOT/*p*TS.^[^ ^62^ ^]^

**Figure 4:**
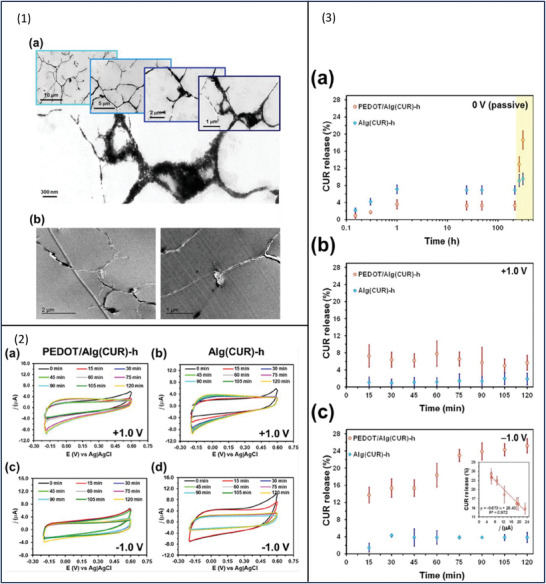
1a,b) TEM images showing PEDOT domains as interconnected spots of nanometric, or micrometric (≈1 µm) molecules embedded in Alg matrix. 2) Cyclic voltammograms recorded at 15 min intervals for (a,c) PEDOT/Alg (CUR)‐h and (b,d) Alg (CUR)‐h electrostimulated by applying a constant voltage of (a,b) +1.0 V or (c,d) −1.0 V. The voltammograms were measured at potential between −0.20 and 0.60 V at a scan rate of 100 mV s^−1^. 3) Release of CUR from PEDOT/Alg (CUR)‐h and Alg (CUR)‐h samples by a) diffusion (passive release) after immersion in an aqueous solution for 9 days and ethanol (pale yellow rectangle) for 4 days and by applying a constant potential of b) +1.0 V or c) −1.0 V for 2 h in aqueous solution. The inset in (c) shows the correlation between the current density (from cyclic voltammograms) and the amount of CUR released from the hydrogel. Reproduced with permission.^[^ ^63^ ^]^ Copyright 2020, American Chemical Society.

Developing drug delivery systems based on hydrophobic drugs remains a challenge; to explore the potential of stimuli‐responsive DDS as a platform to deliver this drug category, curcumin (CUR), a hydrophobic drug, was selected in a study by Puiggali‐Jou and co‐workers.^[^ ^63^ ^]^ CUR‐loaded PEDOT/Alg hydrogel was prepared in two steps; first CUR and alginic acid (AA) were dissolved in ethanol, and after that PEDOT:PSS was added to the mixture. Gelling was reached by adding CaCl_2_ solution to the mixture yielding PEDOT:PSS and alginate (Alg) hydrogel loaded with CUR. In the absence of electrical stimulation, slow and slight release (3%) of CUR was determined due to the hydrophobic nature of the drug and the formation of intermolecular bonds with the matrix. Upon active release, however, 25% CUR was released in 2 h. During electrochemical stimulation, voltage values of either +1.0 or −1.0 V were applied for 2 h on both PEDOT/Alg (CUR) and Alg (CUR). After 1 h of stimulation, the amount of CUR released from PEDOT/Alg (CUR) and Alg (CUR) was 3.6 ± 1.0% and 7.1 ± 1.0%, respectively. The low drug release was linked to the hydrophobicity of CUR, to overcome this issue, the release media was replaced by ethanol. By doing so, the amount of CUR from PEDOT/Alg (CUR) and Alg (CUR) has increased to 12.9 ± 1.7% and 9.1 ± 1.4%, respectively. Negative voltage‐controlled drug release was higher compared to positive or passive release (**Figure** **4**(3)).

Conduction paths were seen in the TEM images (Figure 4 (1)) that explain the electroresponsive behavior of the hydrogel. PEDOT domains were seen as interconnected spots of nanometric, or micrometric (≈1 µm) molecules embedded in the Alg matrix. The CV voltammograms for both PEDOT/Alg (CUR) and Alg (CUR) at voltage values of either +1.0 or −1.0 V are shown in Figure 4 (3). At stimulation of +1.0 V, the voltammograms suggest that the hydrogel response to the stimulation is due to the presence of PEDOT. The corresponding voltammograms of Alg (CUR) at +1.0 and −1.0 V show a minimum reduction of electrochemical activity, indicating that the amount of drug released from this system is caused mainly by ions diffusion rather than as a response to the stimulation.

**Figure 5:**
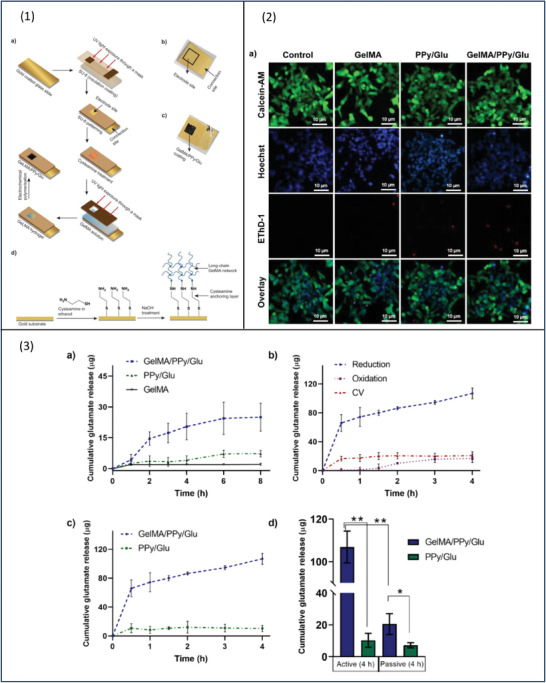
1) Schematic illustration showing a) the process of preparing GelMA/PPy/Glu. b) Electrode site. c) GelMA/PPy/Glu coating. d) Drug incorporation process. 2) Biocompatibility testing. 3a). Cumulative Glu release from GelMA/PPy/Glu, PPy/Glu, and GelMA coatings by passive diffusion in phosphate buffer saline solution (PBS) for 8 h. b) Electrically stimulated release profile of Glu from GelMA/PPy/Glu coatings showing the influence of different electrical triggers, i.e., constant reduction (−0.6 V), constant oxidation (+0.6 V), and cyclic voltammetry (CV) cycle sweeps (−0.6 to + 0.6 V at a rate of 100 mV s^−1^). c) Comparison of cumulative Glu release profile from GelMA/PPy/Gly and PPy/Glu coatings upon constant reduction (−0.6 V). d) Bar graph showing the comparison active and passive release of Glu from GelMA/PPy/Glu and PPy/Glu coatings after 4 h. (Data presented as mean ± SD) (^*^ *p* ≤ 0.05, and ^**^ *p* ≤ 0.01). Reproduced with permission.^[^ ^64^ ^]^ Copyright 2022, Elsevier.

One of the main limitations of PPy is its low drug‐loading capacity. Research into overcoming such issue by Bansal and group resulted in the development of conducting polymer hydrogel (CPH) made of gelatin methacrylate (GelMA) and PPy.^[^ ^64^ ^]^ Using electrochemical polymerization, glutamate (Glu), a model drug, was incorporated into the hydrogel yielding GelMA/PPy/Glu system (**Figure** **5**(1)). GelMA/PPy/Glu extract was used in biocompatibility testing using LIVE/DEAD viability/cytotoxicity assay on undifferentiated SH‐SY5Y neurons. The cells were exposed to the extract for 24 h and the average viability of the control group was 95.4 ± 4.90%. The test groups' results were 94.3 ± 2.94% for the GelMA hydrogel, 91.7 ± 6.27% for PPy/Glu, and 90.0 ± 3.73% for GelMA/PPy/Glu. There was no statistical difference compared to the control group indicating the biocompatibility of the materials (Figure 5 (2)).

CV demonstrated a large increase in charge storage capacity and long‐term electrochemical stability (1000 CV cycles). Glu is an anionic drug and was loaded in both GelMA/PPy/Glu and PPy/Glu via electropolymerization at +0.9 V. The use of constant oxidizing potential makes PPy in the oxidized state which then forms electrostatic interactions with negatively charged Glu. The porous network of GelMA/PPy/Glu allows higher drug loading. Both systems were deposited into a gold electrode (1 cm^2^) to study passive and active drug release profiles. In the absence of stimulation, the amount of Glu released from GelMA/PPy/Glu, PPy/Glu, and GelMA hydrogel were 25.0 ± 6.82, 7.2 ± 1.59, and 2.1 ± 0.53 µg, respectively. To study the influence of the hydrogel, the amount of Glu released from GelMA/PPy/Glu was compared to that from conventional PPy/Glu films, resulting in 14 times higher amounts of Glu released from the hydrogel‐based system.

The release study (Figure 5 (3)) indicates a five times increase in Glu release with electrical stimulation of (−0.6 V) compared to passive release. At constant CV sweeps, the total amount of Glu release was 20.7 ± 5.50 µg over 4 h. Using different types of electrical stimulation namely CV (± 0.6 V at 100 mV s^−1^) and constant oxidation (+0.6 V) has resulted in no significant difference in the amount of drug release (*p* > 0.5). On the other hand, the application of constant reduction potential at −0.6 V has increased the Glu release from GelMA/PPy/Glu compared to that from PPy/Glu, 106.8 ± 7.48 and 7.20 ± 1.59 µg, respectively (Figure 5 (3)). The drug release using a reduction reaction was six times higher than that with oxidation and five times higher than the unstimulated release. Therefore, for negatively charged molecules, the application of negative potential results in higher drug release due to electrostatic repulsion forces. The opposite is true; positive potential leads to the electrostatic attraction between the anionic drug and CP chain and the amount released could be driven by the diffusion process.

A promising application of PANi was recently demonstrated, where when used as wound dressing, both the electrical stimuli and cargo synergistically helped to accelerate wound healing. A hydrogel comprising vitamin D‐loaded PANi/chitosan composite was developed for wound healing. 1% w/v chitosan was dissolved in acetic acid solution and left to homogenize, after that 2% w/v PANi and vitamin D solution in 6:4 v/v water:ethanol was added to chitosan solution. The hydrogel formation was completed by gradually adding sodium TPP solution. The current of the hydrogel was 1455 µA. The hydrogel was tested both in vitro and in vivo; cell culture studies showed that despite the effect of vitamin D as a growth factor, the electrical stimulation itself enhanced cell proliferation. In vivo wound healing studies on Wistar rats confirmed the positive effect of electrical stimulation, however, a more pronounced wound healing effect was obtained with vitamin D (wound healing within 12 days) compared with control, nontreated rats (wound healing within 21 days). The study has compared four different groups for wound healing, group 1 control with no intervention, group 2 used marketed formulation, group 3 blank hydrogel, and group 4 conducting hydrogel. Both group 3 and 4 microscopic images show epithelization and fibroblast migration suggesting the formation of healthy tissues with no scar formation. Compared to group 1, group 2 had relatively faster healing; however, it was slower than groups 3 and 4, and scar tissues were noticed.

A separate study investigating PANi revealed CP can have multiple modes of drug release in hydrogels.^[^ ^65^ ^]^ When PANi was combined with a polyvinyl alcohol (PVA) hydrogel, the mechanism of indomethacin released was a combination of drug migration as a result of the electric field, but also the erosion of the hydrogel, suggesting that CPs can be used to control the process of hydrogel erosion. It is worth remarking that incorporating PANi can reduce drug encapsulation, as reported by,^[^ ^66^ ^]^ which was attributed to the hydrophobic characteristics of PANi. Other findings with PANi include the successful demonstration of in vivo and in vitro biocompatibility electroactive DDS, with tuneable “on‐off” release of indomethacin and dextran, and amoxicillin, respectively.^[^ ^12^, ^67^ ^]^ The latter study demonstrated that 9 “on‐off” pulses can be achieved with a PANi‐blended hydrogel.

Hyaluronic acid based hydrogels were made electroactive when blended with a combination of PANI and reduced graphene oxide (rGO). Hyaluronic acid has been explored for multiple healthcare applications, including both drug delivery and tissue engineering.^[^ ^68^ ^]^ The material displays a range of desirable biomedical features, such as inherent therapeutic effects, tuneable mechanical properties, and biocompatibility.^[^ ^69^ ^]^ Hyaluronic hydrogels are inherently nonconductive, while the addition of either PANI or rGO resulted in conductivity in the order of 10^−6^ S cm^−1^. However, the simultaneous addition of both PANI and rGO appeared to have a synergistic effect with conductivity increasing by one order of a magnitude to 10^−5^ S cm^−1^, where the authors hypothesized that the integration of both conductive materials imparted different conductivity sources in the hydrogel structure. Nonetheless, the hydrogel exhibited sufficient conductivity, where ibuprofen release was triggered by an on‐and‐off voltage stimulus. Furthermore, cumulative ibuprofen release was enhanced with a sustained voltage over 140 min, where the release for 0, 1, and 3 V were 35%, 60% and 86%, respectively.^[^ ^70^ ^]^

### 5.2 Polymeric Films

Polymeric films have garnered significant attention as a novel and adaptable approach to DDS. These thin, flexible sheets composed of biocompatible polymers offer a versatile platform for controlled drug release, allowing for precise dosing and prolonged therapeutic effects.^[^ ^71^ ^]^ Their ease of application and potential for localized treatments make them an attractive option for various medical scenarios. Notably, the integration of CPs into these polymeric films has introduced an exciting dimension to drug delivery.^[^ ^71a^ ^]^ By leveraging the electrical properties of CPs, these films can respond to electric fields, enabling triggered drug release.^[^ ^19^ ^]^

**Figure 6:**
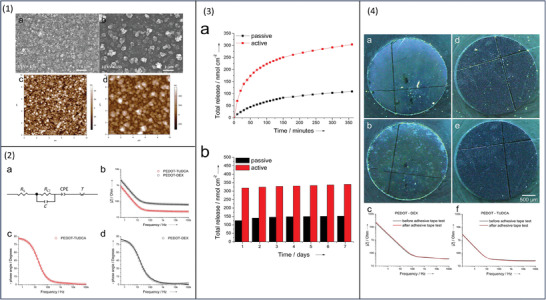
1a,b) Scanning electron microscopy (SEM) images and c,d) atomic force microscopy (AFM) images show granular‐like structures that are commonly associated with electropolymerized polyethylenedioxythiophene (PEDOT). 2a) Illustration of circuit model b–d) bode plot describing the frequency response of both systems that shows frequency‐independent impedance interval from 10^5^ to 10^2^ Hz. before and after the adhesive tape test. 3a,b) Active versus passive cumulated release of tauroursodeoxycholic acid (TUDCA) from PEDOT/TUDCA coated electrodes with respect to different time intervals. 4a–e) Electrochemical impedance spectroscopy (EIS) images of films before and after the adhesive tape test. c,f) The resistive performance of both films before and after the adhesive tape test was in the frequency range of 0.1–100 Hz. Reproduced with permission.^[^ ^72^ ^]^ Copyright 2019, John Wiley and Sons.

Tauroursodeoxycholic acid (TUDCA), a natural bile acid, has attracted interest due to its anti‐inflammatory and neuroprotective activity for the treatment of central nervous system diseases. There is a desire to control the release of TUDCA, which prompted researchers to investigate the electroactive release of the compound. TUDCA was incorporated within PEDOT to form PEDOT/TUDCA film via CV electrodeposition. The study compared PEDOT/TUDCA film to PEDOT/dexamethasone film. In terms of film morphology, both SEM and atomic force microscopy (AFM) images show granular‐like structures that are commonly associated with electropolymerized PEDOT (**Figure** **6**(1)). The charge transport dynamics in both films were tested using electrochemical impedance spectroscopy (EIS) circuit modeling with a frequency interval of 105–0.5 Hz. This is important because freely transporting charge ensures the conductivity of the film, while on the contrary, their transport is hindered by resistance. In circuit modeling, resistance is represented by impedance. When impedance is independent of electrical signal frequency, charge transport along the conductive film is improved. PEDOT showed typical near ohmic behavior leading to an increase in the frequency‐independent impedance interval from 105 to 102 Hz. Based on the resistance values, PEDOT/TUDCA (*R*s≈20 Ω) has higher electrical conductivity compared to PEDOT/dexamethasone (*R*s≈40 Ω) (Figure 6 (2)). There was an initial burst release of TUDCA in both passive and active release methods, however, with the active release, both the drug amount and the release rate were greater. Upon the application of 500 CV cycles (360 min), the drug release was 305 ± 6 nmol cm^−2^, whereas passive release resulted in 109 ± 29 nmol cm^−2^ drug release (Figure 6 (3)). When discontinuing the electrochemical stimulus, its influence on the system may remain for a longer period. To investigate that, TUDCA‐containing electrodes that were subjected to previous electrochemical stimulation have been used to investigate further compound release. The passive release of TUDCA from electrodes subjected to previous stimulation was measured over 7 days and yielded a release amount of 340 ± 44 nmol cm^−2^ compared to 152 ± 45 nmol cm^−2^ for those unprocessed (Figure 6). This confirms that the total active drug release involved both the amount released when an electrochemical stimulus was on, and the amount released afterward. Collectively, they have achieved higher release in comparison to passive drug release. Delamination of CP films due to mechanical stress has been reported to assess that adhesion measurements are performed by test tape. The optical images show the stability of PEDOT/TUDCA film, in contrast, PEDOT/dexamethasone film has randomly scattered patches (Figure 6 (4)). To quantify the extent of lamination before and after the adhesion measurement, EIS was used. The resistive performance of both films was in the frequency range of 0.1–100 Hz, however, the diffusional capacitance *C*LF for PEDOT/TUDCA and PEDOT/dexamethasone was in the order of 5% and 13%, respectively (Figure 6 (4)). The study has compared PEDOT/TUDCA and PEDOT/dexamethasone films since the latter has been previously proposed for neural drug delivery. The viability assays showed that PEDOT/TUDCA has low cytotoxicity and more biocompatibility compared to PEDOT/dexamethasone system, thus, it is suitable as a neural implant.^[^ ^72^ ^]^

**Figure 7:**
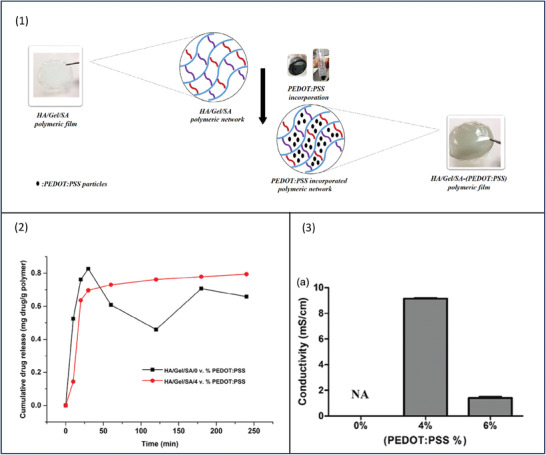
1) Fabrication of hyaluronic acid (HA)/Gel/sodium alginate (SA) polymeric film and the incorporation of polyethylenedioxythiophene (PEDOT):PSS. 2) CIP release data from HA/Gel/SA film and HA/Gel/SA‐(PEDOT:PSS) film. 3) Conductivity data of different concentrations of PEDOT:PSS as 4 and 6% v/v in HA/Gel/SA‐(PEDOT:PSS) films. Reproduced with permission.^[^ ^73^ ^]^ Copyright 2022, John Wiley and Sons.

To study the effect of PEDOT:PSS inclusion and concentration on ciprofloxacin release, the solvent‐casted polymeric films comprising hyaluronic acid/gelatin/sodium alginate (HA/Gel/SA) were developed with different concentrations of PEDOT:PSS (0, 4 and 6% v/v) (**Figure** **7**(1)). To load the drug, 1 cm^2^ film samples were cut and immersed in a ciprofloxacin drug solution.^[^ ^73^ ^]^ The ciprofloxacin release data are shown in Figure 7 (2). When a sustained drug release profile is desired, the uncontrolled burst release results in losing a great amount of the drug in a short period. In this study, compared to the pure HA/Gel/SA films, adding PEDOT:PSS to the formulation resulted in a decrease in the amount of burst release. The filler effect of PEDOT:PSS has retained ciprofloxacin molecules within its polymeric network. The incorporation of PEDOT:PSS has led to better control over the drug release with sustained profile overcoming the issue of initial uncontrollable burst release.

Four‐point resistivity probing equipment was used to measure the electrical conductivity of PEDOT:PSS‐containing films, those containing 4% v/v PEDOT:PSS has higher electrical conductivity (9.2 × 10^−3^ S cm^−1^) compared to those containing 6% v/v (Figure 7 (3)). This is attributed to the uniformity of PEDOT:PSS distribution within the polymeric matrix at lower concentrations allowing continuous movement of electrons, whereas, at a concentration of 6% v/v, the CP molecules agglomerate and reduce electron transport. Although it was presumed that the higher the PEDOT:PSS concentration, the higher the conductivity, this study confirms that the optimum concentration of CP is concentration independent.

**Figure 8:**
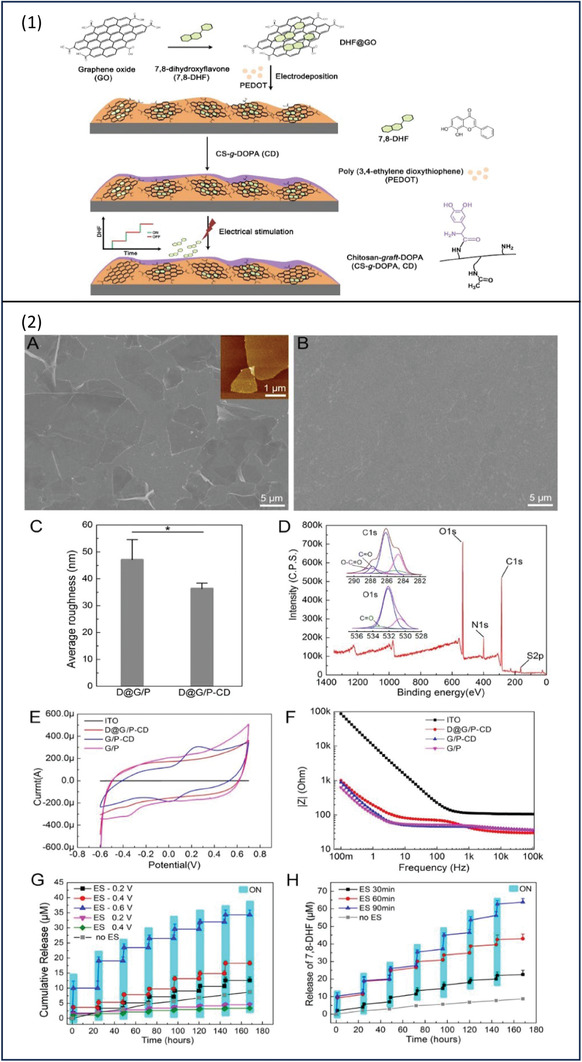
1) schematic illustration of the fabrication process. 2) a) SEM image of D@G/P, the image in the upper right corner was the AFM image of single‐layered GO sheet. b) SEM image of D@G/P‐CD. c) average surface roughness of D@G/P and D@G/P‐CD was determined based on the AFM images. d) XPS analysis verified the oxidative transformation of the catechol functional groups within CD. e) cyclic voltammograms of ITO, G/P, G/P‐CD and D@G/P‐CD recorded in 0.1 M KCl. f) Electrochemical impedance spectroscopy was performed on ITO, G/P, G/P‐CD, and D@G/P‐CD. g,h) Staircase release of 7,8‐DHF from D@G/P‐CD film at different voltages for 60 min per day and at −0.6 V for different duration. Reproduced with permission.^[^ ^75^ ^]^ Copyright 2022, Elsevier.

Sun et al. were able to develop a hybrid film that can provide electrically controlled drug release and deliver exogenous electrical signals to neuronal cells.^[^ ^74^ ^]^ Initially, graphene oxide (GO) nanocomposites were loaded with neuroprotective drug 7,8‐dihydroxyflavone (7,8‐DHF) by π–π stacking, and the drug‐loaded nanocomposites were then deposited inside PEDOT film and finally, to improve the biocompatibility, the film was coated with Dopamine‐*graft*‐Chitosan (CD). The hybrid system was able to deliver different signals such as electrical signals, nanotopographical signals by GO, and the drug to the neural cells resulting in neuronal mitochondrial biogenesis that was confirmed by immunofluorescence staining and gene expression. It has been found that extreme electrical stimulation has damaging effects on the conductive films including delamination and cracking, nevertheless, D@G/P‐CD film was able to retain its structural integrity after 200 release stimulation (**Figure** **8**(2a,b)).

In the absence of electrical stimulation, a minor drug amount was passively released. On the other hand, administering electrical stimulation in the amount of −0.2 V for 90 min has resulted in an increase in drug release to (2 × 10^−6^ m), further, an increase in drug release was achieved by increasing the applied voltage by −0.4, and −0.6 V to ≈4 × 10^−6^ m and ≈8 × 10^−6^ m, respectively. Increasing the duration of stimulation has enhanced drug release, these results prove that the amount of drug release is dependent on both the voltage and duration of the applied stimulus. Applying multiple cycles of on–off type stimulation resulted in a staircase profile with a positive step corresponding to an electrically promoted release (Figure 8(2g–h)). The electrochemical properties of the film were studied via CV at a scan rate of 0.05 Vs^−1^ and were conducted in 0.01 m PBS (pH 7.4) media. By comparing the area under the curve of cyclic voltammograms, the results indicate a high charge to storage capacity for G/P, G/P‐CD, and D@G/P‐CD but not the bare ITO. The characteristic reversible redox reactions seen in the graph confirm that the film is electrochemically active (Figure 8(2e)).

### 5.3 Patches and Microneedles

Patches and microneedles (MNs) have emerged as innovative and patient‐friendly drug delivery systems, offering noninvasive and convenient alternatives to traditional administration methods. Patches, often applied to the skin, provide controlled release of drugs over time, while MNs painlessly breach the skin's surface to deliver therapeutics directly into the bloodstream or underlying tissue.^[^ ^75^ ^]^

**Figure 9:**
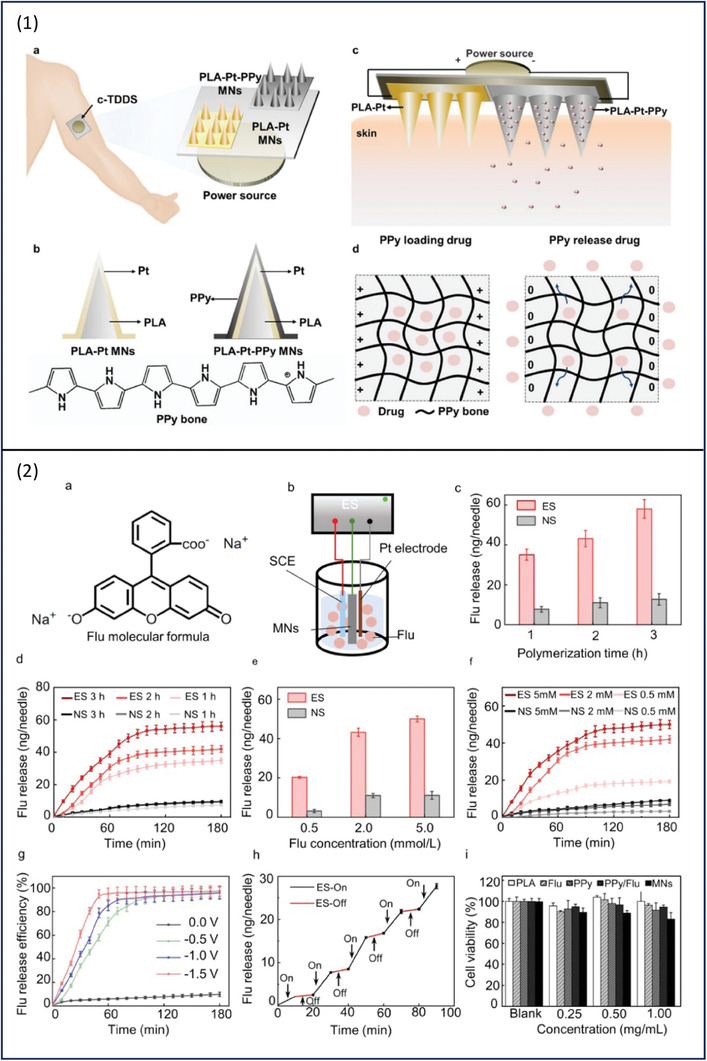
1) a,b) Schematic illustration of the transdermal MNs patch showing the PPy film layer. c,d) Drug loading and release mechanisms. 2) a) The Molecular structure of Flu. b) Schematic diagram of the in vitro Flu release from PLA‐Pt‐PPy MNs using three electrode system. c) The effect of polymerization time on the amount of Flu release. d) Flu release over time. e) The effect of Flu concentration on the amount of Flu release. f) Flu release with different concentrations. g) The release efficiency as a result of voltage alteration. h) Flu release in on/off profile. i) cell viability test. Reproduced with permission.^[^ ^77^ ^]^ Copyright 2022, American Chemical Society.

Yang and co‐workers have developed smart patches for electrically controlled and on‐demand transdermal drug delivery to enhance drug permeation through the skin barrier.^[^ ^76^ ^]^ The system consisted of conductive MNs and a two‐electrode microneedle patch (t‐EMNP); two sets of MNs were developed using polylactic acid platinum (PLA‐Pt) and polylactic acid platinum polypyrrole (PLA‐Pt‐PPy) (**Figure** **9**(1)). Fluorescein was used as a model drug and was loaded on the MNs by polymerization; both polymerization time and fluorescein concentration have influenced the MNs' drug loading capability. Polymerization times of 1, 2, and 3 h were able to achieve drug loading by 35.062 ± 2.753, 43.165 ± 4.058, and 58.03501 ± 2.09456 ng per needle, respectively. Longer periods of electrochemical polymerization result in thicker PPy film and accordingly higher drug loading. Nevertheless, very thick films are not favorable in this application as it covers the tips of MNs preventing their penetration into the skin. The drug loading was influenced by fluorescein concentration which has a direct impact on drug deposition on MNs, fluorescein concentrations of 0.5, 2, and 5 mmol L^−1^ were able to produce drug loading of 20.276 ± 0.524, 43.165 ± 2.058, and 49.985 ± 1.499 ng per needle, respectively. An electrochemical workstation was used to conduct the drug release study; it consisted of two main parts; the three‐electrode system and the working solution which was PBS. The three electrodes were a counter electrode made of platinum wire, a reference electrode comprising SCE electrode, and the test electrode was PLA‐Pt‐PPY MN patch. The fluorescein release was controlled by electrical stimulation which when absent neglected drug release was detected. The release rate was altered by applying different potentials. The thickness of the PPy film, electrical stimulation period, and value of applied potential have controlled the amount of fluorescein release. There was an initial fast release of fluorescein upon electrical stimulation which was followed by an equilibrium state, and about 80–90% of fluorescein was released. However, without electrical stimulation, between 10–20% of the drug was detected which is presented as a result of drug diffusion. Changing the applied voltage has an impact on the drug release efficiency, a potential value of −1.5 V resulted in higher fluorescein release efficiency. When the current output is high, there will be more charges transferred through the circuit and the PPy backbone will be in a reduced state leading to more drug release. Another common test with electrically stimulated formulations is the on‐off test; herein, when the electrical stimulation was on the off mode, there was nearly no drug release. Whereas, with the on mode, fluorescein release presented a linear release profile. The system showed superior electrochemical and mechanical properties and high drug‐loading ability. To further investigate its efficacy of it, fluorescein was replaced with glucocorticoids and tested on atopic dermatitis‐bearing mice and the results showed that infiltration of both inflammatory cells and inflammatory factors was reduced remarkably and mice returned to normal state. PLA‐Pt‐PPy biocompatibility was also investigated in this study using a cytotoxicity test, the data confirmed great biocompatibility with more than 80% cell viability (Figure 9 (2)).

### 5.4 Polymeric Nanoparticles

Polymeric nanoparticles are known for their ability to encapsulate drugs and protect them from degradation, as well as their ability to sustain drug release over a period of time.^[^ ^77^ ^]^ These nanoparticles can be made from various materials, including biodegradable polymers, and can be functionalized with targeting ligands or imaging agents to improve their specificity and efficacy.^[^ ^78^ ^]^ CPs have also been investigated for their potential use in polymeric nanoparticle‐based DDS.^[^ ^79^ ^]^ The unique electrical conductivity of CPs can be utilized to control drug release rates and achieve targeted delivery, making it a promising material for drug delivery applications.

**Figure 10:**
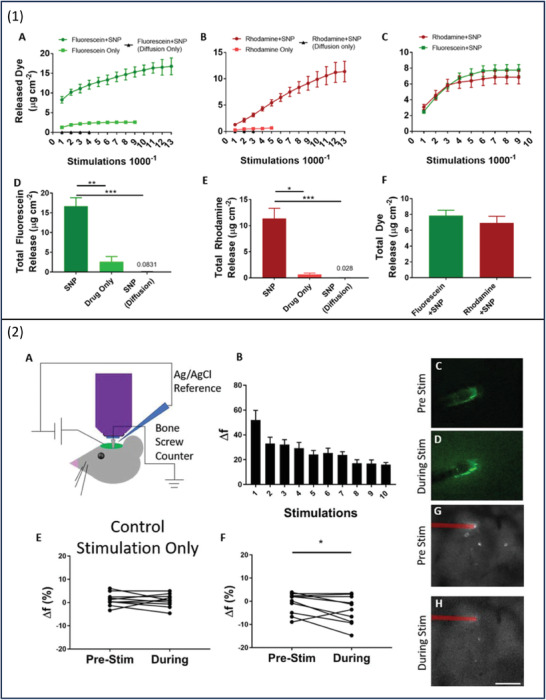
1) A–F) Fluorescein and rhodamine active release from PEDOT/SNP films compared to the passive release, the corresponding analogues, and co‐release of combined compounds SNPs. 2) A) Schematic illustration of the in‐vivo study model. B) The release of Flu using different stimulation values. C, D) Representative images of Flu release before and during stimulation. E, F) The release of Flu using different stimulation values. G, H) DNQX loaded electrode before and during stimulation. Reproduced with permission.^[^ ^81^ ^]^ Copyright 2019, John Wiley and Sons.

In an attempt to enhance the drug loading capacity, silica nanoparticles were processed to form thiol nanoparticles which were further oxidized to form sulfonate nanoparticles (SNPs); the latter was subjected to selective incorporation of hexadecyl trimethylammonium bromide forming nonporous and porous SNPs. The nanoparticles were then doped with PEDOT films. Low drug loading is one of the inherent issues of CPs; herein, the production of PEDOT‐coated nanoparticles has significantly increased drug loading capacity owing to SNPs' high loading capacity (0.55 cc g^−1^). The tested compounds were fluorescein and rhodamine, which are negatively and positively charged, respectively. Aqueous solutions of the individual compounds and a mixture of both were used to suspend the nanoparticles, followed by sonication to enhance the compounds' migration through the pores on the surface of the particles. To trigger the drug release from the PEDOT coatings of nanoparticles, the CV voltage sweep test in the range of 0.8 to −0.6 V was used. The active release of fluorescein and rhodamine from the SNPs was increased by 6.4 and 16.8 times, respectively. The data also shows that upon 5000–9000 stimulations, there was a significant increase in the release of the compound. Compared to the active compound release, the amount of compound detected after passive release by diffusion was negligible. Simultaneous corelease of both drugs was determined upon the electrical stimulation using CV stimulation type. The data showed that the corelease was at a slower rate compared to the release of individual drugs which could be caused by compound interaction (**Figure** **10**(1)).^[^ ^80^ ^]^

A more complex in vivo model was used in this study to further understand the formulation performance (Figure 10(2a)). CV was used in different pulses to initiate the release of fluorescence (Figure 10(2b)), and the results show more controlled release and diffusion of the dye in the surrounding tissues when stimulated compared to pre‐stimulation conditions (Figure 10(2c,d)). Fluorescein was replaced by DNQX, a small and negatively charged compound that inhibits glutamate transmission. Stimulated release in vivo resulted in modulation in neural activity (Figure 10(2e,f)). This work has demonstrated the ability of codelivery of drugs which is of major interest in the area of polypharmacy, leading to better patient adherence. The system's ability to deliver both positively and negatively charged drugs extends its suitability to incorporate a wide range of compounds.

Another study has explored developing PEDOT nanoparticles (PEDOT NPs) aiming to achieve higher sensitivity to the electrical stimulation for controlled and on‐demand release of the payload.^[^ ^81^ ^]^ PEDOT NPs were loaded with pyrimethamine (PYR), a potent pharmacological chaperone used for the treatment of certain rare disorders. The particle size of loaded and unloaded PEDOT NPs were 215 ± 3 and 203 ± 1 nm, respectively; whereas the net surface charges were −26 ± 7 and −29 ± 6 mV for the loaded and unloaded, respectively. The drug loading capacity of PEDOT NPs was 11.4 ± 1.5%. Both passive and active release methods were employed to study the effect of electrical stimulation on PEDOT NPs. In vitro active drug release was tested using two electrical stimulation methods, CV and CA. In general, the test voltage should be in the range that does not change the chemical structure of the drug; for instance, PYR oxidizes at around 1.2 V, and the selected range for this test was −0.50 and 0.50 V. Continuous CV stimulus was applied for 5, 15, and 30 min. Very low drug release was detected in the absence of electrical stimulus, 1.6% in 24 h and 18% in 80 days. Active drug release was achieved using CV with applied voltage from –0.5 V to 0.5 V. In contrast to the passive release, active release was able to release 50% of PYR in 30 min. A CA test using a constant voltage of 1.0 V was explored and the drug release was 35% for the same test duration. Similar time points were used in the CA test with constant voltage at 1.0 V. Compared to CV, CA was able to induce more drug release by 15%. As theoretically anticipated, constant voltage triggers more drug release since it causes compound release only, unlike the dynamic scans employed by CV in which the drug is in constant release and reincorporation processes in the polymer chain. CV voltammograms at different time points exhibited similar shapes and areas. Nevertheless, as the drug release increased with time during the anodic scan, the cathodic charge increased which was indicated by a reduction in the cathodic current density. The release study lasted for 80 days, where there was a burst release for the initial 24 h that was followed by a slow progressive release for 5 days with a percentage release of 4.1%. Only 18% of the drug was released after 80 days. This phenomenon was explained by PYR's low solubility in the aqueous PBS media (0.01 mg mL^−1^). The study concluded that PEDOT NPs offer a promising electroresponsive nanocarrier approach due to ease of synthesis, high stability, low toxicity, and fast response to the stimulus.

Metoprolol, a potent beta blocker, was loaded into PPy nanoparticles that were cross‐linked into PVA polymer networks forming nanocomposite hydrogels. Nanoparticles were prepared by the biocatalytic method after that they were encapsulated in PVA by reticulation with glutaraldehyde. This study compared the drug release from PVA hydrogel and PVA/PPy hydrogels in both passive and active release conditions. With the systems being unstimulated, a slight increase in metoprolol release was seen from PVA/PPy hydrogels. Drug release from PVA hydrogels with and without stimulation has followed a similar pattern due to the nonionic nature of the system. On the other hand, an increase in metoprolol release was detected from PVA/PPy hydrogels upon electrical stimulation. Increasing the concentration of PPy in the hydrogel resulted in more drug release over 360 min. A total of 100% of metoprolol was released from PVA/PPy35 hydrogel. The study emphasized that the release of the drug from a conductive filler is controlled by the CP's intrinsic redox properties, independent of the electromigration of the ionized drug, resulting from the interaction between the CP and the electrical stimulation. The application of constant cathodic potential resulted in higher drug release compared to both passive and anodic potential which is in agreement with previous reports. An anodic potential of + 5 V has resulted in a decrease in drug release by 26% compared to the cathodic potential. This was explained by the generation of positive charges on the CP chains, consequently, negatively charged counterions from the electrolytic medium are incorporated to maintain charge neutrality which leads to blocking drug release.^[^ ^82^ ^]^

### 5.5 Coatings in Drug Delivery Systems

**Figure 11:**
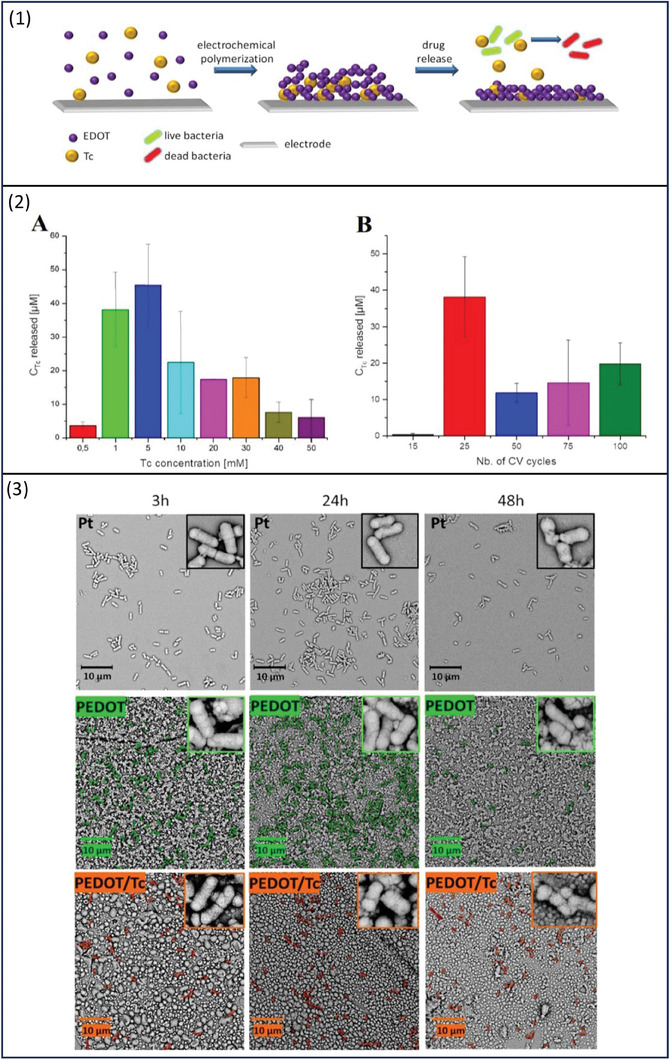
1) Schematic illustration of the electrochemical polymerization and drug release processes. 2) The influence of A) the tetracycline (Tc) concentration and B) number of cyclic voltammetry (CV) cycles on drug release showing maximum release at concentration of 5 × 10^−3^ m and 25 cycles. 3) Antibacterial activities of PT, PEDOT, and PEDOT/Tc at different time points 3, 24, 48 h. Reproduced with permission.^[^ ^84^ ^]^ Copyright 2021, Elsevier.

Bacterial growth on medical implants is a serious issue; one proposed solution is to coat them with an antibacterial layer.^[^ ^83^ ^]^ Czerwinska‐Glowka and team have developed PEDOT polymeric coating containing the antibacterial drug, tetracycline (Tc) at a concentration of 1 × 10^−3^ m by electrochemical polymerization process (**Figure** **11**(1)).^[^ ^84^ ^]^ The drug loading capacity and charge storage capacity were 194.7 ± 56.2 µg cm^−2^ and 19.15 ± 6.09 mC cm^−2^, respectively. Different numbers of CV cycles ranging from 15 to 100 were used to test both the loading and release of Tc from PEDOT/Tc matrices, the data concluded that the maximum drug release was obtained with 25 cycles. Whereas drug loading capacity was limited with few CV cycles; with 50 or more CV cycles, it was hard for the matrix to fully reduce and release Tc. Thus, it is important to choose the number of CV cycles that allow electrochemical degradation of the matrix. The electrochemical properties of PEDOT and PEDOT/Tc matrices were similar. PEDOT/Tc matrices prepared from 1 and 5 × 10^−3^ m showed the maximum Tc release by 38.2 ± 11.03 × 10^−6^ and 45.5 ± 12.2 × 10^−6^ m, respectively (Figure 11 (2)). PEDOT/Tc matrix biological activity was tested against *E. coli*, Gram‐negative bacteria, and compared to PEDOT only matrix. Both matrices exhibited antibacterial activities, however, there was a pronounced enhancement when Tc was incorporated showing a threefold decrease in bacterial growth after 48 h, the results showed a robust antibacterial activity of the developed matrix (Figure 11 (3)). The study has indicated that when electropolymerization method is used for drug incorporation into the polymeric matrix, extending the process time or drug concentration in the media will not have a positive impact on either the drug loading capacity or the drug release.

PBS has been used widely for release studies from CPs due to its physiological relevancy and ability to charge transport. During the CV test, a series of oxidation/reduction reactions occur within the polymeric chain resulting in the capture/release of cargo ions. The polymer volume shrinks when a low potential is applied causing Tc release. UV–Vis spectrophotometry is a commonly used analytical method to quantify electrochemical drug release; the absorption peak of Tc was at 363 nm.

CPs can also be used as a coating to modulate drug release. Coatings offer multiple advantages in drug delivery, including being compatible with a range of drugs, simplicity and immediate release.^[^ ^85^ ^]^ They are also versatile and can be coated onto a wide range of substrates of different geometries and material classes.^[^ ^86^ ^]^ In electroactive drug delivery, PPy loaded with both Dex and a nerve growth factor was electrochemically coated onto neural electrodes. The aim was to enhance the biological functionality over existing electrodes by inhibiting the inflammatory cellular cascade whilst displaying no toxic effect to primary neural cells. The study demonstrated how Dex release can be controlled by CV, sweeping between −0.8 and 0.6 V.^[^ ^87^ ^]^

Other studies have coated TPU with PANI:PSS and demonstrated enhanced insulin release in the presence of voltage^[^ ^88^ ^]^ but also onto a porous titanium substrate, further demonstrating coating's versatility to adapt to different material classes. In the latter study, PPy loaded with Dex was electrochemically deposited onto a titanium implant intended for bone grafting and reported an increase in in vitro Dex release under – 2 V over 24 h.^[^ ^89^ ^]^

Coatings also present with their own challenges, such as delamination and limited drug loading.^[^ ^90^ ^]^ One CP‐based study found that coating can hinder release. Drug‐loaded electrospun fibers were produced and subsequently coated with PPy using a chemical method, where the coating was found to inhibit drug release. Compared to uncoated samples, the coated samples subjected to 0 V resulted in a significant loss in drug release over 225 min. The authors then subjected the coated samples to 0.3, 0.5, and 1 V and reported a significant increase in drug release with 1 V, which was comparable to the uncoated samples, albeit displaying different release kinetics. Understandably, the coating acted as a physical barrier that hindered drug release. Whereas, for the voltage‐enhanced release, the authors postulated that the PPy was oxidized under voltage and caused the coating to become molecularly porous, which subsequently allowed the drug to permeate through. This is indeed a different release mechanism in comparison to other voltage‐stimulated release mechanism but nevertheless demonstrates how varying the voltage can alter drug release.^[^ ^91^ ^]^ Overall, these CP coating studies demonstrate that electroactive coatings can be applied to different substrates, further widening their applicability in drug delivery.

There are some limitations associated with CPs, including that they allow the delivery of only small, charged compounds, low drug loading, insoluble, and difficult to process.^[^ ^92^ ^]^ Although the previously mentioned studies have explored different approaches to overcome CPs drawbacks, there still require further improvements. Thus, strategies that have proven effective in enhancing CPs performance in different fields could be applied to the development of CP‐based DDS.

## 6 Fabrication of CP‐Based Drug Delivery Systems

Aside from their inherent properties, the fabrication process can influence the performance of the electroactive DDS. To date, fabricating electroactive DDS can be achieved by several routes, including drug‐loaded NP, thin films, and hydrogels. Films are the most common options, which can involve coating a substrate with a drug‐loaded CP. These films have been achieved by conventional methods such as solvent casting, dip coating, and drop casting. In addition, and owing to the monomer's intrinsic properties, CP films can be produced by electrochemical polymerization: the process of oxidation of the monomer and the growth of the polymer chain. This process involves placing a substrate, which could simply be the electrode, inside of an electrolyte medium containing the respective CP monomers and subsequently applying a voltage. The process can be performed in either aqueous or nonaqueous solvents. The advantages of electropolymerization over conventional methods are both precision and speed, especially as the time‐consuming process of solvent evaporation is obviated. Moreover, polymerization can be performed directly onto the electrode thus saving more time. Regarding precision, the film thickness can be controlled by tuning the voltage, among other parameters.^[^ ^93^ ^]^ In addition to the thickness, such parameters also influence other film properties. Furthermore, when one considers films of 20 nm thicknesses that can be achieved, electropolymerization appears to be an appealing preparation route for DDS. Moreover, the surface of films can be manipulated to increase surface area and thereby increase drug loading capacity. A larger surface area equates to an increased number of sites for drug incorporation. Another attractive method compatible with CP preparation that can create a variety of different surface morphologies is soft template electropolymerization.^[^ ^94^ ^]^ The appeal of a soft template comes from the need for fewer preparation steps, ease of synthesis, and easy removal of the template.^[^ ^95^ ^]^ This technique has been successfully demonstrated to increase drug‐loading capacity,^[^ ^94b^ ^]^ although its suitability for large‐scale production is yet to be determined.^[^ ^96^ ^]^

The prospect of increasing drug loading has been explored by other fabrication technologies. Three‐dimensional (3D) structures with controlled porosity and substrate geometry can be achieved using 3D printing, which is a collection of emerging technologies that possess a remarkable degree of control over DDS geometry with enhanced digital precision.^[^ ^97^ ^]^ An example is the use of two‐photon polymerization to produce MNs. Two‐photon polymerization is compatible with photopolymers, which currently are inherently nonconductive. Thus, coating the photopolymer substrate with a CP requires an intermediate step, such as coating the MNs with gold, which then allows CP to be electrodeposited onto the substrate. As a proof of concept, an arbitrary needle length and diameter of 500 and 130 µm, respectively, were printed, where the authors proceeded with in vivo studies that revealed the successful transdural release of dexamethasone.^[^ ^98^ ^]^

**Figure 12:**
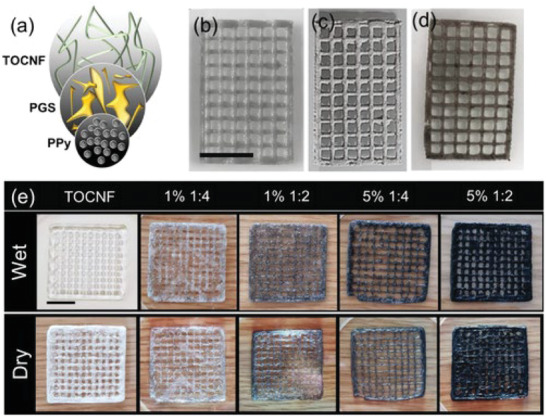
Schematic illustration of drug delivery system based on polypyrrole (PPy) fabricated using direct ink writing showing porous structures with controlled dimensions. Reproduced with permission.^[^ ^101^ ^]^ Copyright 2020, John Wiley and Sons.

Further to two‐photon polymerization, direct ink writing has also been used. Direct ink writing can generate 3D structures without needing temperature, which is suited for this application as some drugs are thermally labile. Moreover, studies have revealed that the conductivity of PEDOT, PPy, and PANi considerably decreased as a function of temperature.^[^ ^99^ ^]^ A further benefit is that like other extrusion‐based 3D printing techniques, direct ink writing can align polymer chains, which has been found to improve conductivity.^[^ ^100^ ^]^ An example of a DDS containing PPy fabricated with direct ink writing is portrayed in **Figure** **12**. Although the study did not investigate the effect of voltage on drug release, it provides further evidence that 3D printing technologies can play a substantial role in fabricating electroactive DDS.

## 7 Opportunities and Challenges

The above demonstrates that CPs can produce an array of different release profiles by seamlessly altering the voltage stimuli, including pulsatile and delayed release. This provides the opportunity for real‐time control of drug release postadministration, hence possessing the ability to adjust the dose to meet the patient's needs. While the technology remains in its infancy, a number of different DDS have already been explored, which can be used as transdermal patches, injectables, and for transcranial delivery, with desirable features including continuous delivery, biodegradable, compatible with a myriad of drugs, and with a wide preparation route, which is collectively encouraging.

Stimuli‐responsive materials are in general gaining research interest and have been demonstrated to provide enhanced precision over nonstimuli responsive counterparts. Examples include pH‐ and thermoresponsive polymers, which utilize the body's varying physiological microenvironment to release the drug loading at specific external stressors. However, variation due to disease states can unpredictably alter the microenvironment, resulting in off‐target release.^[^ ^102^ ^]^ Moreover, storage conditions for thermoresponsive polymers need to be controlled to prevent premature degradation.^[^ ^103^ ^]^ In comparison, the release mechanism of voltage‐responsive polymers is more robust to their microenvironment.

However, CPs have challenges that also need to be addressed. Currently, formulations are compatible with charged drugs, and further research is needed to demonstrate their compatibility with neutrally charged drugs, which in turn will greatly widen their clinical application. Similarly, further research is needed in expanding their drug loading capacity, where high drug loading (>90% w/w) has yet to be obtained. Increasing the drug loading can help to maintain the system's responsiveness by ensuring that there is sufficient drug available to respond to the trigger and extend the system's longevity. The following criteria must also be met: large therapeutic window as high drug concentrations are anticipated when locally delivered, short half‐life to avoid drug accumulation, and the drug electrochemical degradation must not occur at the applied voltages to the system.

Aside from PEDOT, PPy, and PANi, there are several challenges with using other CPs that restrict their use in drug delivery. Their nature and the mechanism by which they may react to stimulus and release their load remains unknown and thus requires further research. Other CPs are not biocompatible, which could cause adverse reactions or toxicity, and thus may require additional components to isolate them from coming into contact with living tissues. Additional drawbacks such as poor solubility and limited stability have been reported which may significantly impact their effectiveness as DDS.^[^ ^104^ ^]^ Finally, their limited options and synthesis complexity add extra cost and require additional research.^[^ ^18b^ ^]^ Nevertheless, if these drawbacks can be addressed then more CPs will be available. The recent progress made with PEDOT, PPy, and PANi demonstrates their potential value as DDS and warrants further work. Developing new CPs suitable for DDS requires a multidisciplinary approach, merging expertise in organic chemistry, pharmaceutical sciences, and electronic engineering to realize the potential of CPs for medicines. Furthermore, CPs can benefit from enabling technologies, such as 3D printing and artificial intelligence, which are digitalized tools known to accelerate developments.^[^ ^105^ ^]^

Beyond acting as matrices for drug loading, their ability to be digitally controlled could see CPs integrated into electronic devices, such as wearable medicines.^[^ ^106^ ^]^ Furthermore, CP‐based formulations can serve multiple functions. For instance, CPs have successfully been researched as biosensors for drug monitoring,^[^ ^107^ ^]^ and hence there is potential for a DDS act as a theranostic device, simultaneously serving as both drug releasing vehicles and biosensing. Other features of CPs are also exciting and have the potential to transform DDS into a multifaceted system, including energy harvesting and electroporation.^[^ ^108^ ^]^ Beneficially, CPs are extensively explored in other domains, where developments therein can be repurposed to DDS development.

The ability of CPs to be integrated with other electronics has the potential to facilitate an Internet of Things (IoT) infrastructure.^[^ ^109^ ^]^ IoT is a technology that allows siloed products to communicate with each other, providing real‐time data for fast and improved clinical decision making.^[^ ^110^ ^]^ It requires healthcare products to connect to electronic devices, which CPs are capable of achieving.^[^ ^111^ ^]^ From a manufacturing perspective, the use of one material serving multiple functions could reduce costs. Moreover, polymers are cheaper to process than metals, which are the current standard for the electrodes used in biosensors. From a patient usability perspective, the comparatively low density of polymers and the inert properties of polymers are appealing.

## 8 Conclusion

On‐demand drug release is one of the main research directions of DDS. With the aid of various stimulus‐responsive materials such as CPs, research has shown that it is possible to regulate drug release through electrical stimuli. Recent progress into CPs is promising, where researchers were able to develop systems capable of achieving a range of release profiles with both small molecules and peptides, as well as the potential for codelivery. However, there are challenges in utilizing CPs other than PEDOT, PPy, and PANi in drug delivery that need to be addressed to offer opportunities for clinical translation. The use of electrical stimulation DDS is particularly promising due to its ability to easily integrate with sensors or microchips and precisely control the timing and location of drug release. Moreover, there is growing interest in developing self‐powered devices, which has led to increased attention on the self‐powering capabilities of electrical stimulation DDS. Considering that this is an emerging drug delivery approach, further and in‐depth research is needed in the future.

## Conflict of Interest

The authors declare no conflict of interest.

## References

[1] a) Strategies to Modify the Drug Release from Pharmaceutical Systems (Ed.: M. L. Bruschi ), Woodhead Publishing, Sawston, Cambridge, 2015;

[2] R. Sharma , D. Singh , P. Gaur , D. Joshi , Drug Delivery Transl. Res. 2021, 11, 1878. 10.1007/s13346-020-00876-4 33447941

[3] S. Wang , R. Liu , Y. Fu , W. J. Kao , Expert Opin. Drug Delivery 2020, 17, 1289. 10.1080/17425247.2020.1788541 32619149

[4] S. Adepu , S. Ramakrishna , Molecules 2021, 26, 5905. 34641447 10.3390/molecules26195905 PMC8512302

[5] E. R. Gillies , Isr. J. Chem. 2020, 60, 75.

[6] a) F. Li , Y. Qin , J. Lee , H. Liao , N. Wang , T. P. Davis , R. Qiao , D. Ling , J. Controlled Release 2020, 322, 566; 10.1016/j.jconrel.2020.03.051 32276006

[7] a) S. A. Abouelmagd , N. H. A. Ellah , B. N. A. E. Hamid , in Stimuli Responsive Polymeric Nanocarriers for Drug Delivery Applications (Eds.: A. S. H. Makhlouf , N. Y. Abu‐Thabit ), Woodhead Publishing, Sawston, Cambridge 2019;

[8] a) M. Wei , Y. Gao , X. Li , M. J. Serpe , Polym. Chem. 2016, 8, 127;

[9] a) Q. Zhang , G. Kuang , W. Li , J. Wang , H. Ren , Y. Zhao , Nano‐Micro Lett. 2023, 15, 44; 10.1007/s40820-023-01018-4 PMC9908819 36752939

[10] H.‐J. Huang , Y.‐L. Tsai , S.‐H. Lin , S.‐H. Hsu , J. Biomed. Sci. 2019, 26, 73. 31623607 10.1186/s12929-019-0571-4 PMC6798433

[11] A. Zhang , K. Jung , A. Li , J. Liu , C. Boyer , Prog. Polym. Sci. 2019, 99, 101164.

[12] C. J. Pérez‐Martínez , S. D. Morales Chávez , T. del Castillo‐Castro , T. E. Lara Ceniceros , M. M. Castillo‐Ortega , D. E. Rodríguez‐Félix , J. C. Gálvez Ruiz , React. Funct. Polym. 2016, 100, 12.

[13] X. Sun , S. Agate , K. Salem , L. Lucia , L. Pal , ACS Appl. Bio Mater. 2021, 4, 140. 10.1021/acsabm.0c01011 35014280

[14] C. Boehler , Z. Aqrawe , M. Asplund , 2019, 2, 89.

[15] H. Palza , P. A. Zapata , C. Angulo‐Pineda , 2019, 12, 277. 10.3390/ma12020277 PMC6357059 30654487

[16] S. M. Mirvakili , R. Langer , Nat. Electron. 2021, 4, 464.

[17] A. Raza , T. Rasheed , F. Nabeel , U. Hayat , M. Bilal , H. Iqbal , Molecules 2019, 24, 1117. 30901827 10.3390/molecules24061117 PMC6470858

[18] a) A. Puiggalí‐Jou , L. J. Del Valle , C. Alemán , J. Controlled Release 2019, 309, 244; 10.1016/j.jconrel.2019.07.035 31351927

[19] C. Boehler , F. Oberueber , M. Asplund , J. Controlled Release 2019, 304, 173. 10.1016/j.jconrel.2019.05.017 31096016

[20] S. Shah , M. Firlak , S. Berrow , N. Halcovitch , S. Baldock , B. Yousafzai , R. Hathout , J. Hardy , Materials 2018, 11, 1123. 29966387 10.3390/ma11071123 PMC6073109

[21] K. Krukiewicz , A. Kruk , R. Turczyn , Electrochim. Acta 2018, 289, 218.

[22] Kenry , B. Liu , Biomacromolecules 2018, 19, 1783. 29787260 10.1021/acs.biomac.8b00275

[23] J. Kolosnjaj‐Tabi , L. Gibot , I. Fourquaux , M. Golzio , M.‐P. Rols , Adv. Drug Delivery Rev. 2019, 138, 56. 10.1016/j.addr.2018.10.017 30414494

[24] a) H. Machrafi , F. Iermano , S. Temsamani , I. Bobinac , C. S. Iorio , Sci. Rep. 2022, 12, 22107; 36543823 10.1038/s41598-022-26546-8 PMC9772303

[25] a) A. L. Pang , A. Arsad , M. Ahmadipour , Polym. Adv. Technol. 2021, 32, 1428;

[26] E. N. Zare , P. Makvandi , B. Ashtari , F. Rossi , A. Motahari , G. Perale , J. Med. Chem. 2020, 63, 1. 31502840 10.1021/acs.jmedchem.9b00803

[27] H. Zengin , A. Ozer , G. Zengin , Opt. Mater. 2023, 141, 113903.

[28] G. Cao , H. Cui , L. Wang , T. Wang , Y. Tian , ACS Appl. Electron. Mater. 2020, 2, 2750.

[29] a) B. S. Hudson , Materials 2018, 11, 242;29415419

[30] F. Bekkar , F. Bettahar , I. Moreno , R. Meghabar , M. Hamadouche , E. Hernáez , J. L. Vilas‐Vilela , L. Ruiz‐Rubio , Polymers 2020, 12, 2227. 32998386 10.3390/polym12102227 PMC7601494

[31] a) C. S. Day , Á. Rentería‐Gómez , S. J. Ton , A. R. Gogoi , O. Gutierrez , R. Martin , Nat. Catal. 2023, 6, 244; 10.1038/s41929-023-00925-4 PMC11546168 39525327

[32] a) L. Xu , Y. Yang , Y. Mao , Z. Li , 2022, 7, 2100055;

[33] a) Y. Liu , X. Zhang , C. Xiao , B. Liu , Mater. Today Bio 2023, 20, 100668; 10.1016/j.mtbio.2023.100668 PMC10232914 37273791

[34] X. Lin , X. Wu , X. Chen , B. Wang , W. Xu , Int. J. Pharm. 2021, 602, 120591. 33845152 10.1016/j.ijpharm.2021.120591

[35] N. K. , C. S. Rout , RSC Adv. 2021, 11, 5659. 35686160 10.1039/d0ra07800j PMC9133880

[36] a) M. Girtan , R. Mallet , M. Socol , A. Stanculescu , Mater. Today Commun. 2020, 22, 100735;

[37] J. Ouyang , Displays 2013, 34, 423.

[38] C. Boehler , M. Asplund , J. Biomed. Mater. Res., Part A 2015, 103, 1200. 10.1002/jbm.a.35252 PMC4342763 24912825

[39] a) S. Miar , J. L. Ong , R. Bizios , T. Guda , Front. Chem. 2021, 9, 599631; 33614599 10.3389/fchem.2021.599631 PMC7892451

[40] A. Sardar , P. S. Gupta , AIP Conf. Proc. 2018, 1953, 030020.

[41] H. Oyanagi , M. Tokumoto , T. Ishiguro , M. Ogasawara , K. Funahashi , K. Iwata , Synth. Met. 1987, 18, 59.

[42] a) J. Yang , G. Choe , S. Yang , H. Jo , J. Y. Lee , Biomater. Res. 2016, 20, 31; 27708859 10.1186/s40824-016-0078-y PMC5043520

[43] a) Z.‐B. Huang , G.‐F. Yin , X.‐M. Liao , J.‐W. Gu , Front. Mater. Sci. 2014, 8, 39;

[44] a) R. V. Badhe , A. Godse , A. Shinkar , A. Kharat , V. Patil , A. Gupta , S. Kheur , Turk. J. Pharm. Sci. 2021, 18, 483; 34496555 10.4274/tjps.galenos.2020.44452 PMC8430405

[45] a) X. Feng , Y. Zhang , Z. Yan , N. Chen , Y. Ma , X. Liu , X. Yang , W. Hou , J. Mater. Chem. A 2013, 1, 9775;

[46] P. Zarrintaj , M. K. Yazdi , M. Jouyandeh , M. R. Saeb , in Fundamentals and Emerging Applications of Polyaniline (Eds: M. Mozafari , N. P. S. Chauhan ), Elsevier, Oxford 2019.

[47] Y. Park , J. Jung , M. Chang , 2019, 9, 1070.

[48] D. Svirskis , J. Travas‐Sejdic , A. Rodgers , S. Garg , J. Controlled Release 2010, 146, 6. 10.1016/j.jconrel.2010.03.023 20359512

[49] M. Bansal , A. Dravid , Z. Aqrawe , J. Montgomery , Z. Wu , D. Svirskis , J. Controlled Release 2020, 328, 192. 10.1016/j.jconrel.2020.08.051 32877745

[50] G. Kaur , R. Adhikari , P. Cass , M. Bown , T. Gunatillake , RSC Adv. 2015, 5, 37553.

[51] C. A. R. Chapman , E. A. Cuttaz , J. A. Goding , R. A. Green , Appl. Phys. Lett. 2020, 116, 10501.

[52] C. M. Wells , M. Harris , L. Choi , V. P. Murali , F. D. Guerra , J. A. Jennings , J. Funct. Biomater. 2019, 10, 34. 31370252 10.3390/jfb10030034 PMC6787590

[53] a) W.‐W. Yang , E. Pierstorff , J. Lab. Autom. 2012, 17, 50; 22357608 10.1177/2211068211428189

[54] D. Uppalapati , B. J. Boyd , S. Garg , J. Travas‐Sejdic , D. Svirskis , Biomaterials 2016, 111, 149. 27728814 10.1016/j.biomaterials.2016.09.021

[55] S. Paramshetti , M. Angolkar , A. Al Fatease , S. M. Alshahrani , U. Hani , A. Garg , G. Ravi , R. A. M. Osmani , Pharmaceutics 2023, 15, 1204. 37111689 10.3390/pharmaceutics15041204 PMC10145001

[56] P. M. George , D. A. LaVan , J. A. Burdick , C.‐Y. Chen , E. Liang , R. Langer , Adv. Mater. 2006, 18, 577.

[57] A. Bordbar‐Khiabani , M. Gasik , Int. J. Mol. Sci. 2022, 23, 3665. 35409025 10.3390/ijms23073665 PMC8998863

[58] a) J. Li , D. J. Mooney , Nat. Rev. Mater. 2016, 1, 16071; 29657852 10.1038/natrevmats.2016.71 PMC5898614

[59] a) J. Xu , Y.‐L. Tsai , S.‐H. Hsu , Molecules 2020, 25, 5296;33202861

[60] a) M. A. Bhat , R. A. Rather , A. H. Shalla , Synth. Met. 2021, 273, 116709;

[61] C. Kleber , K. Lienkamp , J. Rühe , M. Asplund , Adv. Healthcare Mater. 2019, 8, 1801488. 10.1002/adhm.201801488 30835957

[62] E. Cheah , M. Bansal , L. Nguyen , A. Chalard , J. Malmström , S. J. O'carroll , B. Connor , Z. Wu , D. Svirskis , Acta Biomater. 2023, 158, 87. 36640949 10.1016/j.actbio.2023.01.013

[63] A. Puiggalí‐Jou , E. Cazorla , G. Ruano , I. Babeli , M.‐P. Ginebra , J. García‐Torres , C. Alemán , ACS Biomater. Sci. Eng. 2020, 6, 6228. 33449669 10.1021/acsbiomaterials.0c01400

[64] M. Bansal , B. Raos , Z. Aqrawe , Z. Wu , D. Svirskis , Acta Biomater. 2022, 137, 124. 34644612 10.1016/j.actbio.2021.10.010

[65] T.‐S. Tsai , V. Pillay , Y. E. Choonara , L. C. Du Toit , G. Modi , D. Naidoo , P. Kumar , Polymers 2011, 3, 150.

[66] A. Pourjavadi , M. Doroudian , Polymer 2015, 76, 287.

[67] J. Qu , Y. Liang , M. Shi , B. Guo , Y. Gao , Z. Yin , Int. J. Biol. Macromol. 2019, 140, 255. 31421175 10.1016/j.ijbiomac.2019.08.120

[68] a) K. N. How , W. H. Yap , C. L. H. Lim , B. H. Goh , Z. W. Lai , Front. Pharmacol. 2020, 11, 1105; 32848737 10.3389/fphar.2020.01105 PMC7397973

[69] a) Q. Xu , J. E. Torres , M. Hakim , P. M. Babiak , P. Pal , C. M. Battistoni , M. Nguyen , A. Panitch , L. Solorio , J. C. Liu , Mater. Sci. Eng., R 2021, 146, 100641; 10.1016/j.mser.2021.100641 PMC8409465 34483486

[70] D. Aycan , F. Karaca , A. Koca , N. Alemdar , Int. J. Biol. Macromol. 2023, 231, 123297. 36646353 10.1016/j.ijbiomac.2023.123297

[71] a) B. V. F. Riccio , A. L. P. Silvestre , A. B. Meneguin , T. D. C. Ribeiro , A. B. Klosowski , P. C. Ferrari , M. Chorilli , AAPS PharmSciTech 2022, 23, 269; 36171494 10.1208/s12249-022-02414-6

[72] S. Carli , G. Fioravanti , A. Armirotti , F. Ciarpella , M. Prato , G. Ottonello , M. Salerno , A. Scarpellini , D. Perrone , E. Marchesi , D. Ricci , L. Fadiga , Chem. – Eur. J. 2019, 25, 2322. 30537238 10.1002/chem.201805285

[73] D. Aycan , N. Dolapçı , Ö. G. Karaca , N. Alemdar , J. Appl. Polym. Sci. 2022, 139, e52761.

[74] X. Sun , L. Li , Z. Tan , J. Li , Y. Hou , X. Wang , B. Liu , X. Xing , L. Rong , L. He , J. Controlled Release 2022, 343, 482. 10.1016/j.jconrel.2022.02.002 35134461

[75] a) M. Avcil , A. Çelik , Micromachines 2021, 12, 1321; 34832733 10.3390/mi12111321 PMC8623547

[76] Y. Yang , B. Z. Chen , X. P. Zhang , H. Zheng , Z. Li , C. Y. Zhang , X. D. Guo , ACS Appl. Mater. Interfaces 2022, 14, 31645. 35790212 10.1021/acsami.2c05952

[77] S. Sur , A. Rathore , V. Dave , K. R. Reddy , R. S. Chouhan , V. Sadhu , Nano‐Struct. Nano‐Objects 2019, 20, 100397.

[78] a) M. J. Mitchell , M. M. Billingsley , R. M. Haley , M. E. Wechsler , N. A. Peppas , R. Langer , Nat. Rev. Drug Discovery 2021, 20, 101; 33277608 10.1038/s41573-020-0090-8 PMC7717100

[79] D. Samanta , N. Hosseini‐Nassab , R. N. Zare , Nanoscale 2016, 8, 9310. 27088543 10.1039/c6nr01884j

[80] K. M. Woeppel , X. S. Zheng , Z. M. Schulte , N. L. Rosi , X. T. Cui , Adv. Healthcare Mater. 2019, 8, e1900622. 10.1002/adhm.201900622 PMC6842062 31583857

[81] H. Enshaei , A. Puiggalí‐Jou , N. Saperas , C. Alemán , Soft Matter 2021, 17, 3314. 33629701 10.1039/d1sm00036e

[82] A. M. Orduño Rodríguez , C. J. Pérez Martínez , T. Del Castillo Castro , M. M. Castillo Ortega , D. E. Rodríguez Félix , J. Romero García , Polym. Bull. 2019, 77, 1217.

[83] a) X. Chen , J. Zhou , Y. Qian , L. Zhao , Mater. Today Bio 2023, 19, 100586; 10.1016/j.mtbio.2023.100586 PMC9988588 36896412

[84] D. Czerwińska‐Glówka , W. Przystaś , E. Zabłocka‐Godlewska , S. Student , B. Cwalina , M. Łapkowski , K. Krukiewicz , Mater. Sci. Eng., C 2021, 123, 112017. 10.1016/j.msec.2021.112017 33812635

[85] a) D. Kapoor , R. Maheshwari , K. Verma , S. Sharma , P. Ghode , R. K. Tekade , in Drug Delivery Systems (Ed.: R. K. Tekade ), Academic Press, San Diego, CA 2020;

[86] A. Barik , N. Chakravorty , in Trends in Biomedical Research, (Ed.: M. Pokorski ), Springer International Publishing, Cham 2020.

[87] G. Tian , D. Yang , C. Chen , X. Duan , D.‐H. Kim , H. Chen , ACS Biomater. Sci. Eng. 2023, 9, 5015. 37489848 10.1021/acsbiomaterials.3c00593

[88] R. Morarad , W. Naeowong , A. Sirivat , Drug Delivery Transl. Res. 2023. 10.1007/s13346-023-01399-4 37566363

[89] C. Wu , X. He , Y. Zhu , W. Weng , K. Cheng , D. Wang , Z. Chen , Colloids Surf., B 2023, 222, 113016. 10.1016/j.colsurfb.2022.113016 36427406

[90] E. J. Tobin , Adv. Drug Delivery Rev. 2017, 112, 88. 10.1016/j.addr.2017.01.007 28159606

[91] M. Nasari , D. Semnani , S. Amanpour , Int. J. Polym. Mater. Polym. Biomater. 2023, 72, 1009.

[92] R. Balint , N. J. Cassidy , S. H. Cartmell , Acta Biomater. 2014, 10, 2341. 24556448 10.1016/j.actbio.2014.02.015

[93] A. L. Pang , A. Arsad , M. Ahmadipour , Polym. Adv. Technol. 2021, 32, 1428.

[94] a) Y. Zhao , B. Liu , L. Pan , G. Yu , Energy Environ. Sci. 2013, 6, 2856;

[95] a) Y. Xie , D. Kocaefe , C. Chen , Y. Kocaefe , J. Nanomater. 2016, 2016, 2302595;

[96] J. Liu , T. Yang , D.‐W. Wang , G. Q. Lu , D. Zhao , S. Z. Qiao , Nat. Commun. 2013, 4, 2798.

[97] a) P. Lakkala , S. R. Munnangi , S. Bandari , M. Repka , Int. J. Pharm.: X 2023, 5, 100159; 36632068 10.1016/j.ijpx.2023.100159 PMC9827389

[98] J. Huang , N. Yap , M. Walter , A. Green , C. Smith , J. Johnson , R. Saigal , ACS Biomater. Sci. Eng. 2022, 8, 1544. 35294162 10.1021/acsbiomaterials.1c01305

[99] a) A. Kassim , Z. B. Basar , H. N. M. E. Mahmud , J. Chem. Sci. 2002, 114, 155;

[100] a) A. Schultheiss , A. Revaux , A. Carella , M. Brinkmann , H. Zeng , R. Demadrille , J.‐P. Simonato , ACS Appl. Polym. Mater. 2021, 3, 5942;

[101] R. Ajdary , N. Z. Ezazi , A. Correia , M. Kemell , S. Huan , H. J. Ruskoaho , J. Hirvonen , H. A. Santos , O. J. Rojas , Adv. Funct. Mater. 2020, 30, 2003440.

[102] C. M. Wells , M. Harris , L. Choi , V. P. Murali , F. D. Guerra , J. A. Jennings , J. Funct. Biomater. 2019, 10, 34. 31370252 10.3390/jfb10030034 PMC6787590

[103] E. Lacroce , F. Rossi , Expert Opin. Drug Delivery 2022, 19, 1203. 10.1080/17425247.2022.2078806 35575265

[104] S. Iqbal , S. Ahmad , J. Ind. Eng. Chem. 2018, 60, 53.

[105] a) Y. Abdalla , M. Elbadawi , M. Ji , M. Alkahtani , A. Awad , M. Orlu , S. Gaisford , A. W. Basit , Int. J. Pharm. 2023, 633, 122628; 36682506 10.1016/j.ijpharm.2023.122628

[106] A. Kar , N. Ahamad , M. Dewani , L. Awasthi , R. Patil , R. Banerjee , Biomaterials 2022, 283, 121435. 35227964 10.1016/j.biomaterials.2022.121435

[107] E. Er , A. K. Ates , Microchem. J. 2023, 189, 108534.

[108] M. N. Hasan , S. Sahlan , K. Osman , M. S. Mohamed Ali , Adv. Mater. Technol. 2021, 6, 2000771.

[109] S. J. Trenfield , A. Awad , L. E. Mccoubrey , M. Elbadawi , A. Goyanes , S. Gaisford , A. W. Basit , Adv. Drug Delivery Rev. 2022, 182, 114098. 10.1016/j.addr.2021.114098 34998901

[110] S. Selvaraj , S. Sundaravaradhan , SN Appl. Sci. 2019, 2, 139.

[111] E. W. Zaia , M. P. Gordon , P. Yuan , J. J. Urban , 2019, 5, 1800823.

